# Effect of the habitat and tusks on trunk grasping techniques in African savannah elephants

**DOI:** 10.1002/ece3.11317

**Published:** 2024-04-19

**Authors:** Pauline Costes, Julie Soppelsa, Céline Houssin, Grégoire Boulinguez‐Ambroise, Camille Pacou, Patrick Gouat, Raphaël Cornette, Emmanuelle Pouydebat

**Affiliations:** ^1^ Adaptive Mechanisms and Evolution (MECADEV) UMR 7179 CNRS/MNHN Paris France; ^2^ Institut de Systématique, Evolution, Biodiversité (ISYEB), UMR 7205, CNRS, Muséum National d'Histoire Naturelle, SU, EPHE, UA Paris France; ^3^ Department of Evolutionary Anthropology Duke University Durham North Carolina USA; ^4^ Laboratoire d'Éthologie Expérimentale et Comparée E.R. 4443 Université Sorbonne Paris Nord Villetaneuse France

**Keywords:** food manipulation, *Loxodonta africana*, muscular hydrostat, prehension, Proboscidea, skill

## Abstract

Among tetrapods, grasping is an essential function involved in many vital behaviours. The selective pressures that led to this function were widely investigated in species with prehensile hands and feet. Previous studies namely highlighted a strong effect of item properties but also of the species habitat on manual grasping behaviour. African savannah elephants (*Loxodonta africana*) are known to display various prehensile abilities and use their trunk in a large diversity of habitats. Composed of muscles and without a rigid structure, the trunk is a muscular hydrostat with great freedom of movement. This multitasking organ is particularly recruited for grasping food items while foraging. Yet, the diet of African savannah elephants varies widely between groups living in different habitats. Moreover, they have tusks alongside the trunk which can assist in grasping behaviours, and their tusk morphologies are known to vary considerably between groups. Therefore, in this study, we investigate the food grasping techniques used by the trunk of two elephant groups that live in different habitats: an arid study site in Etosha National Park in Namibia, and an area with consistent water presence in Kruger National Park in South Africa. We characterised the tusks profiles and compared the grasping techniques and their frequencies of use for different foods. Our results show differences in food‐grasping techniques between the two groups. These differences are related to the food item property and tusk profile discrepancies highlighted between the two groups. We suggest that habitat heterogeneity, particularly aridity gaps, may induce these differences. This may reveal an optimisation of grasping types depending on habitat, food size and accessibility, as well as tusk profiles.

## Graphical abstract

We investigate the food grasping techniques used by the trunk of two elephant groups that live in different habitats: an arid study site in Etosha National Park in Namibia, and an area with consistent water presence in Kruger National Park in South Africa. We characterised the tusk profiles and compared the grasping techniques and their frequencies of use for different habitats and tusk profiles. Our results show differences in food‐grasping techniques between the two groups. These differences are related to the habitat and tusk profile differences highlighted between the two groups. We suggest that habitat heterogeneity, particularly aridity gaps, may induce these differences. This may reveal an optimisation of grasping types depending on habitat, food size and accessibility, as well as tusk profiles.

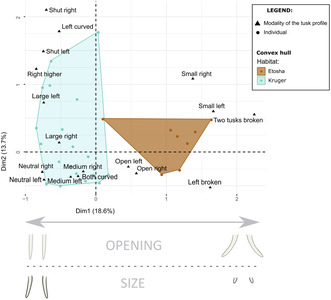

## 1 INTRODUCTION

Grasping, the action of taking an item to hold, move and/or use it, is a crucial function in tetrapods. It is involved in various tetrapod behaviours such as feeding, locomotion, reproduction and social interactions (Pouydebat et al., 2022; Sustaita et al., 2013). Numerous studies have investigated the determinants of manual grasping specifically. It has been shown in various tetrapod species, especially in primates and rodents, that the techniques (i.e., all processes performed to grasp) used for manual grasping vary according to the item properties (Pouydebat, Fragaszy, et al., 2014). Both the size and mobility of the object grasped (e.g., food item) affect grasping techniques in various primate species (bonobos: Bardo et al., 2015; olive baboon: Boulinguez‐Ambroise et al., 2020; strepsirrhines: Peckre et al., 2016; Peckre, Fabre, et al., 2019; Peckre, Lowie, et al., 2019; orangutan, macaque, capuchin, gorillas, chimpanzees: Pouydebat et al., 2009, 2010, 2011, 2014; Brunon et al., 2014; grey mouse lemur: Reghem et al., 2011; Toussaint et al., 2015) and rodent species (rats: Ivanco et al., 1996; Whishaw & Coles, 1996). Relationships between grasping performance and morphological parameters, such as the dimensions of limb segments, have been quantified in grey mouse lemurs too (Boulinguez‐Ambroise et al., 2019; Thomas et al., 2016), humans, bonobos, gorillas and orangutans (Bardo et al., 2018). It is also known that food handling techniques can differ between different groups of chimpanzees and groups of capuchins, certainly as a result of interplay between genetic, social and environmental factors (Humle & Matsuzawa, 2001; Ottoni & Izar, 2008).

Animals can also display precise grasping abilities without having prehensile forelimbs (Pouydebat et al., 2022; Sugasawa et al., 2021). For example, Octopuses use their muscular hydrostat tentacles, i.e. composed only of muscles that retain their volume for any change in shape, to grasp their food with different extension, wrapping and rotation movements (Flash & Zullo, 2023). The three elephants species too can perform various grasping behaviours without prehensile forelimbs, extensively using their trunk (Dagenais et al., 2021; Hart et al., 2001; Racine, 1980; Schulz et al., 2021; Schulz, Reidenberg, et al., 2023; Soppelsa et al., 2022; Wu et al., 2018). Elephant trunk grasping abilities are particularly interesting as the trunk is also a muscular hydrostat allowing it a high degree of freedom of movement (Kier & Smith, 1985). Elephants can extend, bend and twist their trunks, as well as modulate its pressure, allowing them to hold heavy loads, as well as grasp with great precision objects as small as one square centimetre. The trunk is also used to finely manipulate objects, such as peeling a banana (Kaufmann et al., 2023). These abilities are the result of a high sensitivity through unique sensory innervation and whiskers, especially at the trunk tip (Deiringer et al., 2023; Purkart et al., 2022; Rasmussen & Munger, 1996), as well as numerous muscles, tendons and wrinkles (Kaufmann et al., 2022; Longren et al., 2023; Schulz, Reveyaz, et al., 2023). This highly sensitive multitasking organ is involved in a wide repertoire of behaviour such as feeding, watering, environmental exploration, vocalisation, social behaviour, tool making and tool use (Fowler & Mikota, 2008; Haakonsson & Semple, 2009; Hart et al., 2001; Lo Preti & Beccai, 2023; Rasmussen & Munger, 1996; Shoshani, 1998; Yang et al., 2006). Several studies on captive elephants have demonstrated that grasping techniques during feeding are related to the quantity of the item grasped and its properties, such as structure and size (Dagenais et al., 2021; Lefeuvre et al., 2020; Racine, 1980; Schulz et al., 2021, 2022; Soppelsa et al., 2022; Wu et al., 2018). Objects measuring 1–5 cm sideways, like grapes or corn, are usually pinched, i.e. grasped between the fingertips, while larger food items such as melons are wrapped (i.e. with a coiling of the trunk around the object; Racine, 1980). The most common and precise grasping movements performed with the trunk are associated with foraging behaviours (Nair, 1989).

Yet, concerning food grasping and manipulation techniques differences between groups, in elephants and African savannah elephants especially (*Loxodonta africana*), the different data on food grasping was only collected ex‐situ. Our study is the first to quantify in this specie the differences in grasping techniques and their frequency of use between two groups with in‐situ experiments. African savannah elephants are especially known to live in different groups in a large diversity of habitats ranging from montane forests, Miombo and Mopane woodlands, savannah and grasslands to arid deserts, but also a wide altitudinal range from mountain slopes to ocean beaches (Gobush et al., 2021). They are regularly found in 23 countries, over a wide latitudinal range between the desert in Mali and the southern temperate zones in South Africa (Gobush et al., 2021). There is therefore a diversity of habitats between different groups throughout the African continent. Several regional morphological variations, potentially related to the habitat, have been described in different groups of the same elephant species (*L. africana*), regarding the tusks and ears (Hanks, 1972; Steenkamp et al., 2007). The African savannah elephants' diet also varies greatly between groups and habitats. *L. africana* has a generalist herbivorous diet (Owen‐Smith & Chafota, 2012) consisting mainly of grasses, lianas and plants, and sometimes termite mounds, soil deposits and salt‐rich waterholes for mineral supplements (Mwangi et al., 2004; Weir, 1969). During the dry season, African savannah elephant diet also includes leaves and bark (Codron et al., 2006; Estes, 1999) and during the wet season, the proportion of grass in its diet increase, when individuals are less restricted by water availability (Codron et al., 2006; Sukumar, 2003; Van Aarde et al., 2006). Depending on the habitat, African savannah elephants can be primarily grazers or browsers (Sach et al., 2019).

Moreover, the foraging behaviours that involve trunk grasping movements in African savannah elephants can also involve occasionally the use of the tusks (Bielert et al., 2018). These extended on the outside of the mouth upper incisor may indeed be used by the elephants for rooting, bark stripping and food cleaning by hitting it over the tusk (Bielert et al., 2018; Capstick, 1977). Thus, the tusks' characteristics (i.e., tusk profile) might be also an intrinsic factor explaining potential variations in trunk grasping techniques during foraging. Furthermore, given their frontal position, positioned on each side of the trunk, variations in the profile of the tusks can represent a factor limiting the trunk's range of movement. In African elephants, tusks are known to vary in terms of size, presence and breaking between different elephant groups, which could be related to several differences such as the poaching pressure or the habitat (Bielert et al., 2018; Campbell‐Staton et al., 2021; Chiyo et al., 2015; Elder, 1970; Layser & Buss, 1985).

In the present study, we investigate the different trunk grasping techniques in African savannah elephants in‐situ in the context of foraging, and whether the frequency of use of these techniques vary between groups living in different habitats. We realised field observations of two groups living in two different locations in Africa: (1) an area with consistent water presence in Kruger National Park in South Africa and, (2) a drier study site in Etosha National Park in Namibia. The two elephant groups selected for our study are also known to vary regarding tusk profile, with more broken tusks in Etosha elephants compared to the long tusks of Kruger's elephants (Steenkamp et al., 2007; Whyte & Hall‐Martin, 2018).

We predict that (1) trunk grasping techniques will differ between the two groups investigated, according to habitat differences. More precisely, as grass, scrubs and trees are smaller and less profuse in Etosha habitat compared to Kruger habitat, due to differences in water availability (Huang et al., 2022). Yet, several studies in captivity demonstrated that the food items' quantity and size are related to the trunk grasping technique in elephants, with smaller items mostly pinched, and taller and numerous items mainly wrapped (Dagenais et al., 2021; Lefeuvre et al., 2020; Racine, 1980; Wu et al., 2018). We thus predict that the Etosha elephants would mainly use the pinch while Kruger elephants would mostly use the wrap. Moreover, as several studies on Asian elephants (*Elephas maximus*) and captive African savannah elephants demonstrated a majority of right bias in food prehension (Giljov et al., 2017, 2018; Keerthipriya et al., 2015; Lefeuvre et al., 2022; Revathe et al., 2020), we predicted that the studied elephants would also demonstrate a majority of right bias, with no differences between the two habitats. Regarding the tusks, in the early 2000s, the elephants from Etosha and Kruger habitats were known to have low frequencies of tusklessness (Raubenheimer, 2000; Steenkamp et al., 2007). The Kruger elephants were also known to have fewer broken tusks compared to Etosha elephants (1.64% broken tusks in Kruger and 44.4% broken tusks in Etosha, Steenkamp et al., 2007), and thus longer tusks (Whyte & Hall‐Martin, 2018). Therefore, we supposed that (2) in both habitats, the tusklessness would be still quite low, and the breaking and size differences found between the two habitats would be similar to those reported in the literature. Finally, we hypothesised that (3) the presence of tusks, especially the ones facing the trunk, would interfere with the full use of the proboscis, as they would make lateral movements and wrap more difficult.

## 2 MATERIALS AND METHODS

### 2.1 Study areas

We conducted the data collection in two habitats, at the end of the wet season which brings high hygrometry and temperatures and thus lush vegetation. The two habitat descriptions can be found in the Table 1.

**TABLE 1:** Descriptions of the two study areas characteristics and data collects.

Park	Etosha National Park	Kruger National Park
Country	Namibia	South Africa
Surface of the park	22,935 km^2^	20,000 km^2^
Average annual rainfall	358 mm	Between 375 and 500 mm
Average temperature maximum	35°C	35°C
Landscape	Arid to semi‐arid flat savannah	Gallery forests and open grasslands
Vegetation	Sparse and low woody vegetation (mopane trees)	Woody vegetation (mopane trees)
Elephant population	2800	17,000
Elephant density	0.153 Elephants per km^2^	39 Elephants per km^2^
Data collect period	January 2020	February 2020
Film locations	Between Aus and Olifantsbad	Nearby small artificial waterholes and near the Pioneer Dam

First, we carried out this study on African savannah elephants during January 2020 in the Etosha National Park. This animal sanctuary is located in north‐central Namibia in the Kunene region. Central Etosha received an average annual rainfall of 358 mm (Okaukuejo station, 1954–2020), with most rain falling between January and February. The average monthly temperature maximum recorded just south of Etosha Park is 35°C in January–April (2012–14). This protected area corresponds to an arid to semi‐arid savannah with woody vegetation (De Villiers & Kok, 1988). The individuals were filmed between Aus and Olifantsbad in three different locations: Aus which is a very open water body (Figure S1A), a mud point near Aus and along the road between Olifantsbad and the mud point. This woodland area, south of the Etosha Pan and extending westwards, is bushveld dominated by mopane trees. The soil is a calcrete litho sol. The herb stratum is poorly developed because of the calcrete, generally in a coarsely fractured form when superficial. These extensive grasslands are grazed extensively during the rainy season by large numbers of herbivores. During the long dry season, the animals are forced to concentrate near small waterholes, mostly along the southern edge of the Pan, and these regions become severely overgrazed (Le Roux et al., 1988).

The remaining data were collected during February 2020 in the gallery forests and open grasslands of the Kruger National Park in South Africa. Average rainfall varied between 375 and 500 mm per year (Shingwedzi station, 1977–2018). We collected the data in the north of the park in Mopane forests and bushes (Kos et al., 2012; Venter et al., 2003). The woody vegetation of this landscape is dominated by mopane shrubs, but 1–2 m in height. The soils in this landscape structurally consist of flat to concave plains with several marshes. The geological rock formations upon which this landscape developed are basalt and the soils usually have a high clay content (Gertenbach, 1983). As water is present throughout the park and elephant feeding methods are more diverse near water (Abraham et al., 2021; Tehou et al., 2019), we collected behavioural data mostly nearby small artificial waterholes and near the Pioneer Dam. We filmed the vast Dam (Figure S1B) from the Mopani rest camp, in an elevated location, approximately 20 m, to cover a large area. For the waterhole and grassland areas, we filmed from the road, over a distance up to 15 km from the rest camp.

An illustration of the landscapes of study sites in Etosha and Kruger parks is available in Figure S1. To make this article easier to read, we have used ‘Etosha or Kruger elephants’ instead of ‘specimens observed at the study sites in Etosha or Kruger parks’.

### 2.2 Conditions of field data collection

To avoid human disturbance, we took the data at a distance with the elephants and from human areas (camps and roads). The data were taken in video format, at approximately 100 m from the elephants near the Pioneer Dam and an average of 30 m with the videos from the road. The 100 m represents the distance imposed between the camp and the Pan, where the elephants showed no behaviour directed at the observers. We followed the CNRS guidelines and European Union regulations (Directive 2010/63/EU). We recorded with Sony FDR‐AX 53 cameras that have a 4K image quality and a ×30 zoom, during the morning and at the end of the afternoon when the elephants are the most active (Rozen‐Rechels et al., 2020). The videos enable observational accuracy on the brief trunk movements (Revathe et al., 2020). Each elephant encounter was filmed when the elephant performed grasping with a visible trunk. Moreover, most of the videos include several individuals, from 2 to 41 elephants. In this case, the behaviour of each elephant was analysed individually, to avoid individual selection and to maximise the number of elephants recorded. The tracked animals were filmed continuously until they left the view.

### 2.3 Elephant identification and tusks profile establishment

**FIGURE 1:**
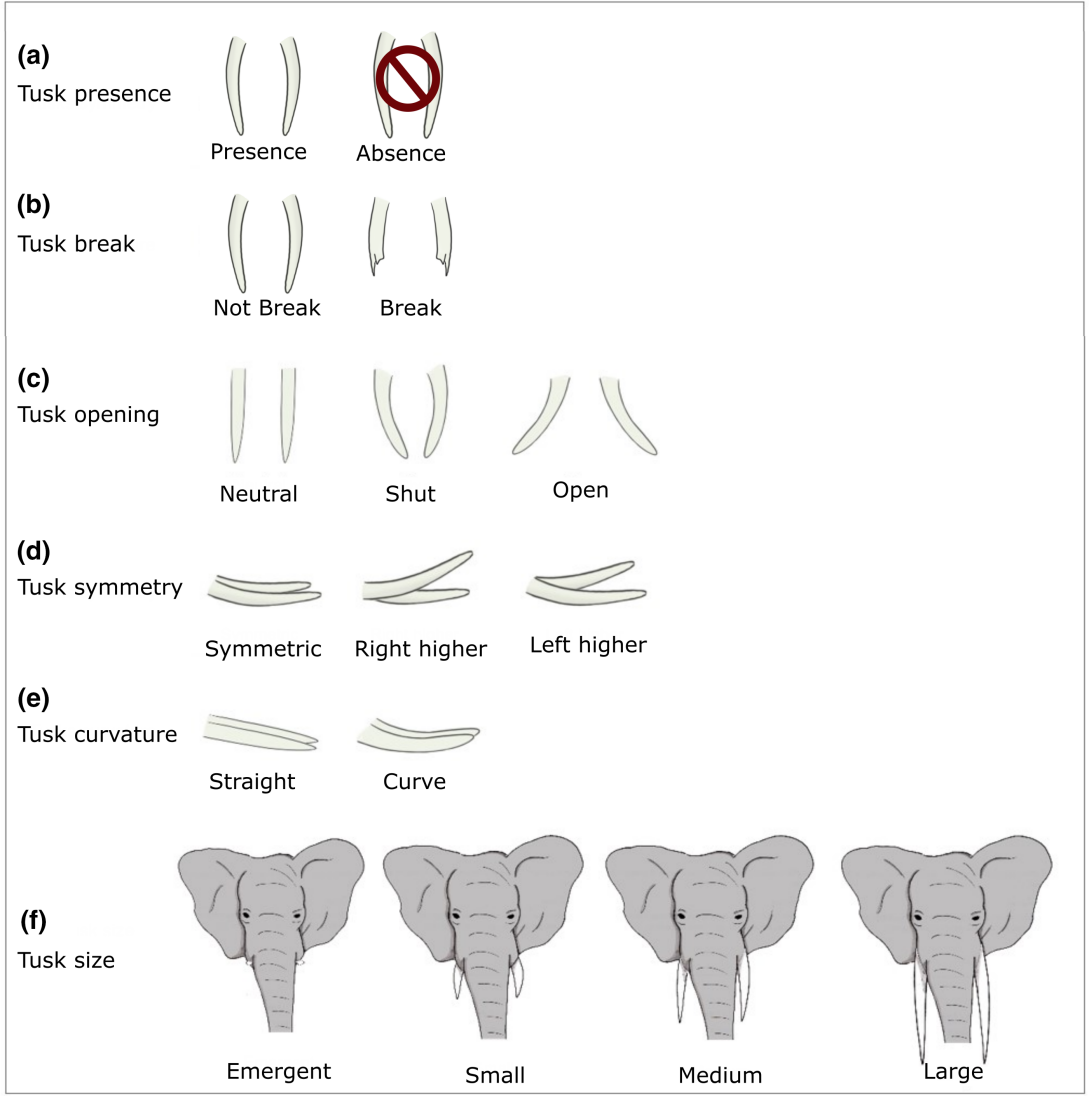
Illustrations of the observed elephant tusks presence, breaking, symmetry, opening, curvature and size modalities. The illustrations represent both tusks for better understanding, however each tusk has been studied independently.

The individuals were identified by two persons (JS, PC) through the videos and the information were corroborated. We identified each individual by checking the following combination of physical characteristics: ear shapes, ear holes and veins, wart shapes or back shapes (Moss, 1996; Webber & Lee, 2020). We also used the tusk characteristics as indicators for recognition. Only the elephants identified with certainty have been kept in the dataset. For each elephant, both tusks were described in detail which allowed us to establish their tusks profiles (illustrations of the tusk details in Figure 1; Vidya et al., 2014). These tusk profiles were used to define, for each tusk, the tusk presence or lack (Figure 1a), its potential break or not (Figure 1b), and its opening (Figure 1c), in other words, if the tusk tip was pointed at the trunk or towards the outside, or stayed straight. We noted too if both tusks were symmetrical or if one was higher (Figure 1d), when the elephant is perfectly in profile, with the head straight, regardless of the position of the trunk. We also looked for the curvature of the tusk (Figure 1e), a binary variable: any tusk that is not completely straight is considered to be curve. Finally, we defined the size of the tusk with four levels (Figure 1f): emergent tusk, small, medium and large. We estimated tusk length relative to the animal head height. The head height is the distance from tear gland to lip line (Black et al., 2019). We classified a tusk as Large if the length of the tusk was more than the head height. We classified it as Medium if the tusk length was between the head height and half head height, Small if it was less than the half head height. We classified a tusk as Emergent if it was visible but its length was less than quarter head height. The length of the tusks was assessed visually, but no measurements were taken. A second observer confirmed the classification made by the first.

The observed elephants were also sex and age‐classified. The sex was determined based on their genitals, breasts, shoulder height, body shape, head shape, tusk shape and group composition (Moss, 1996; Poole, 1994). The age class was determined using physical characteristics as the body shape, the tusk size or absence, the shoulder height, breast size, which could mean that the elephant has already had a calf (Moss, 1996; Shrader et al., 2006). The group composition was also used, i.e. calf presence or solitary specimen (Moss & Poole, 1983). We used four age classes: infant (0–4 years), juvenile (5–9 years), sub‐adult (10–15 years), adult (15+ years) (Lee & Moss, 1995; Moss, 2001). The sex and age class of all individuals were verified by two observers.

We, therefore, identified 64 individual African savannah elephants with the combination of the criteria of sex, tusks profile and particular physical characteristics. Of those elephants identified, 48, of which 38% are females and 62% are males, were from Kruger habitat and 15, of which 45% are females and 55% are males, were from Etosha habitat. Among the 64 observed elephants, 34 performed the grasping task (Table S1). Only males were included in some analyses (described in Section 2.5). Of the elephants studied at Etosha, 20% were infants, 0% juveniles, 13% sub‐adults and 67% adults. Of the elephants studied at Kruger, 4% were infants, 8% juveniles, 11% sub‐adults and 77% adults. The number of the elephants used in the several analyses of this study is detailed in Tables 2 and 3.

**TABLE 2:** The distribution of the elephants in the analyses of grasping technique and tusk profiles.

	Analyses	Habitats	Individuals (*N*)
Grasping technique/habitat	GLMM and Boxplots	Etosha	Between 5 and 12
		Kruger	Between 7 and 22
Tusk profile/habitat	Bar plots and Fisher's exact test	Etosha	10
		Kruger	36
	Correlation matrix and MCA & PERMANOVA	Etosha	8
		Kruger	31

**TABLE 3:** The distribution of the elephants in the analyses of the grasping technique according to the tusks profile.

Analyses	Tusks profile variables	Modalities	Individuals (*N*)
GLMM and Boxplots	Breaking	No tusk break	7
		Both tusk break	3
		Left break	1
		Right break	1
	Opening	Open	Between 8 and 16
		Neutral	Between 1 and 3
		Shut	3
	Presence	Tuskless	3
		Both tusks	8
		Right tusk	1

### 2.4 Grasping, techniques and items

We explored the most commonly observed feeding grasp during the fieldwork. This is when the elephants grasped food items with their trunk to put them in their mouth directly. Furthermore, we noted the food items that were grasped. The three food items considered in this study that are grasped by both groups are grass, small branches (diameter below 2 cm) and leaves.

**FIGURE 2:**
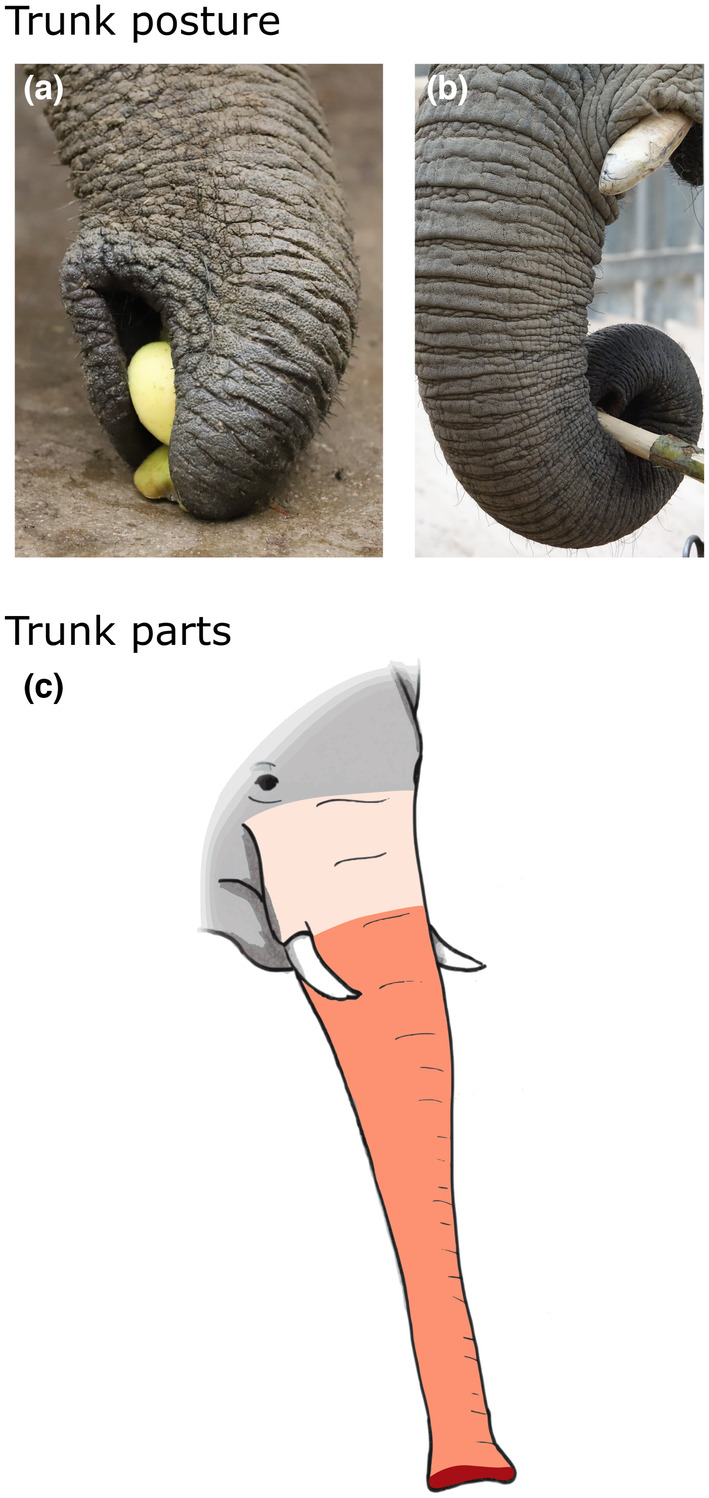
Illustration of the criteria of the trunk grasping techniques. The first criterion is the grasping type, in particular the trunk posture: the pinch with the fingers of the trunk tip, sometimes helped with a suction (a) and the wrap of the trunk around an item (b). Pictures by R. Cornette and J. Soppelsa. The second criterion is the trunk parts used in the movements represented in schematic form: the base, the shaft and the tip (c).

Finally, we used two criteria to define trunk grasping techniques. The first criterion is the grasping type (Figure 2), which possesses two dependent modalities: the trunk posture, i.e. the pinch with the fingers of the trunk tip or the wrap (Figure 2a,b respectively; Lefeuvre et al., 2020), and the trunk parts used in the movements (Figure 2c). We slice the trunk into three parts, from proximal to distal: the base, shaft and tip (Schulz, Reveyaz, et al., 2023). The second criterion is grasping direction bias, which possesses two dependent modalities: lateral or frontal direction to grasp an item and also right or the left side to grasp from the elephant's perspective (Giljov et al., 2017; Haakonsson & Semple, 2009; Keerthipriya et al., 2015; Lefeuvre et al., 2022; Martin & Niemitz, 2003).

### 2.5 Data analyses

We analysed each video and noted, for every grasping occurrence, the focus elephant with its sex, age class, tusks' characteristics and the habitat where the animal was observed. We also noted the item involved, the grasping type, i.e. trunk posture and trunk parts used in the movements (specifically the base and tip of the trunk), and the grasping direction bias (lateral vs. fontal and right vs. left). 1.5 months per person were needed to encode all the videos and extract 1732 food grasping and detailed tusk profiles.

All statistics were done on the R version 4.1.1 software (R Core Team, 2021), *p*‐values under .05 being considered as statistically significant. In order to study the relationships between trunk technique, habitat and modalities of the tusk profile, generalised linear mixed models (GLMMs) were used and the results are illustrated using boxplots. To compare the tusk profiles between the two habitats a correlation test, a multiple correspondence analyses (MCA), a permutational multivariate analysis of variance (PERMANOVA) and Fisher's exact tests were used and the results are illustrated using biplot and bar plots.

### 2.6 Generalised linear mixed model

To evaluate the effects of the habitat and the item grasped on the frequency of use of different trunk grasping techniques, we performed GLMM. We built the models using the binary modalities of the trunk technique (trunk posture, base and tip of the trunk uses, grasping direction bias: lateral vs. fontal and right vs. left) as dependant variable, the habitat (Etosha or Kruger), the food item grasped (grass, leaves or small branch) and the interaction between them as fixed factors, and the individuals, their sex and age class as random factors. A binomial distribution was used to analyse the dependent variables as the data were binary. Models were built using the package glmmTMB (Brooks et al., 2017) and assumptions of the models (under/overdispersion) were tested in the DHARMa package (Hartig, 2018).

**FIGURE 3:**
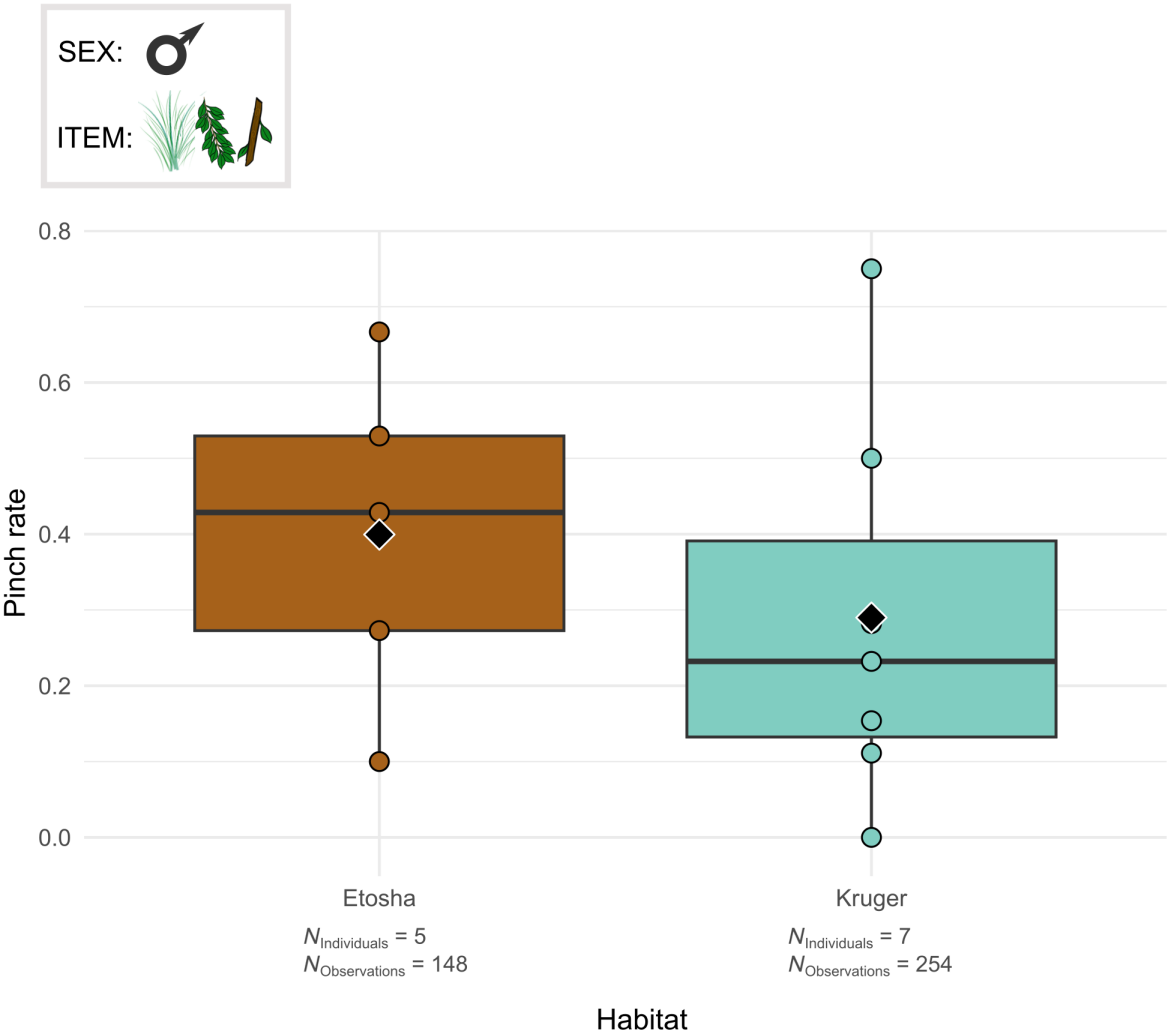
Boxplots of the pinch rate per male individual according to the habitat. The grasped food items were the grass, leaves and small branches. The box at the top left indicates the individuals studied (here, males) and the items considered (here, grass, leaves and small branches). Black diamonds indicate the average pinch rate for each habitat. Chi‐squared test: *p* < .05.

**FIGURE 4:**
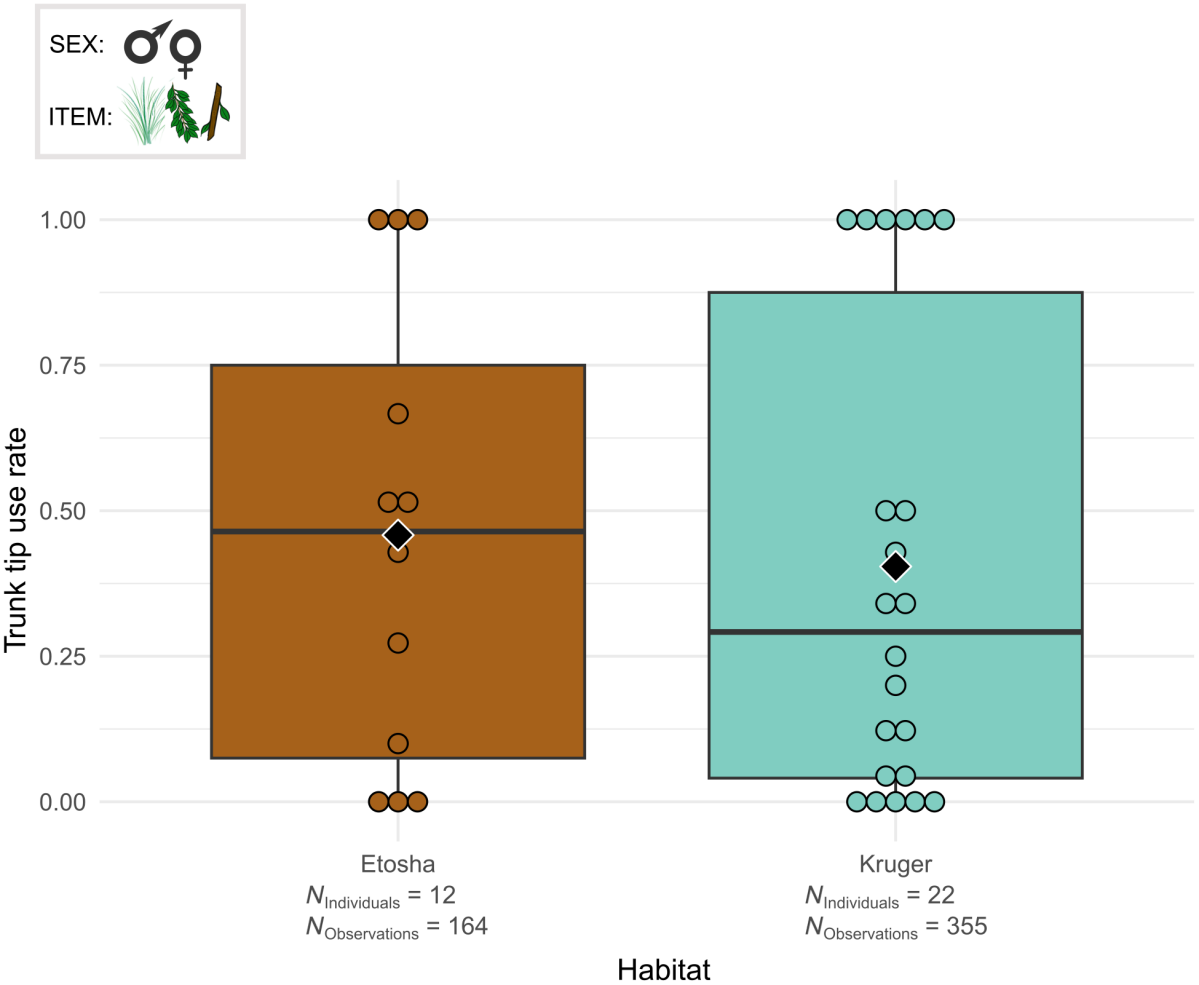
Boxplots of the tip grasp rate per male and female individual according to the habitat. The grasped food items were grass, leaves and small branches. Black diamonds indicate the average trunk tip use rate for each habitat. Chi‐squared test: *p* < .001.

**FIGURE 5:**
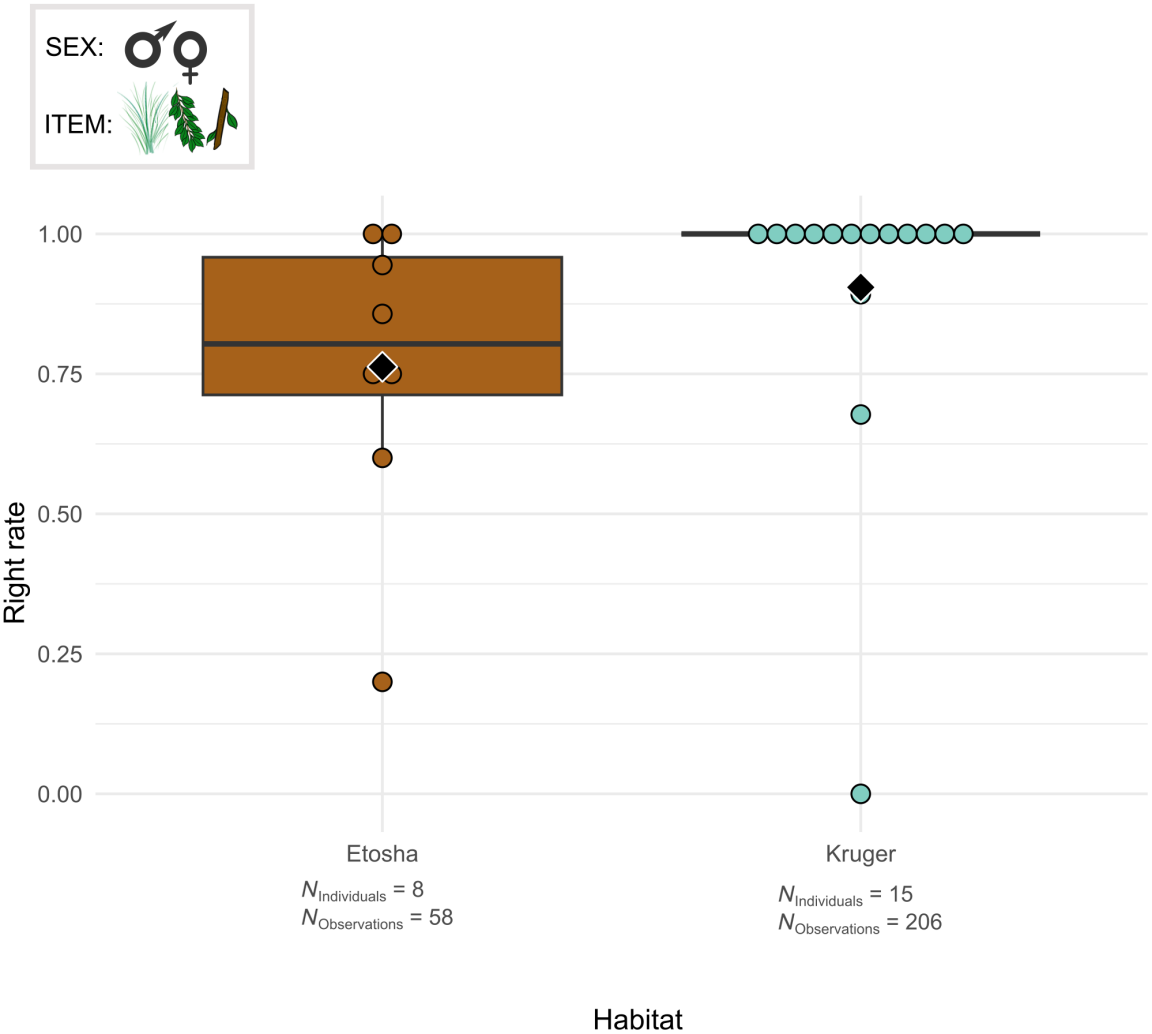
Boxplots of the right grasp rate per male and female individual according to the habitat. The grasped food items were the grass, leaves and small branches. Black diamonds indicate the average right rate for each habitat. Chi‐squared test: *p* < .05.

We used the following protocol to select the model: we tested the model including all data, i.e. males and females, and the grasping for the three food items (grass, leaves and small branches). If one of the DHARMa tests was non‐significant, we tested the model including only males (the sex most represented in our data) and the grasping for the three items. If one of the DHARMa tests was again non‐significant, we tested the model including both sexes but only grass grasping (the most represented food item in our data). If one of the DHARMa tests was again non‐significant, we tested the model including only males and only grass grasping. Finally, if one of the DHARMa tests was again non‐significant, we considered the model to be false and did not present its results in the article (details of the models used are presented in Table S2). On the contrary, when the assumptions of the models are validated using DHARMa tests, we ran Type I sum of squares using, the ‘Anova’ function of the ‘stats’ package (Chambers & Hastie, 1992), to compares the models in sequential order. Detailed results of Type I sum of squares are available in Table S2. We also compared the grasping techniques between modalities of the tusk profile using GLMM with the same protocol. Each GLMM giving significant results was illustrated using boxplots (Figures 3, 4, 5 and 9, 10, 11, 12, 13). As the individuals do not all have the same number of observations, we used percentages in order not to bias the visualisations. To do this, the percentage of use of the different modalities of the variables of the grasping technique was calculated for each individual and used to create boxplots. So, each dot represented an elephant.

### 2.7 Correlation matrix

**FIGURE 6:**
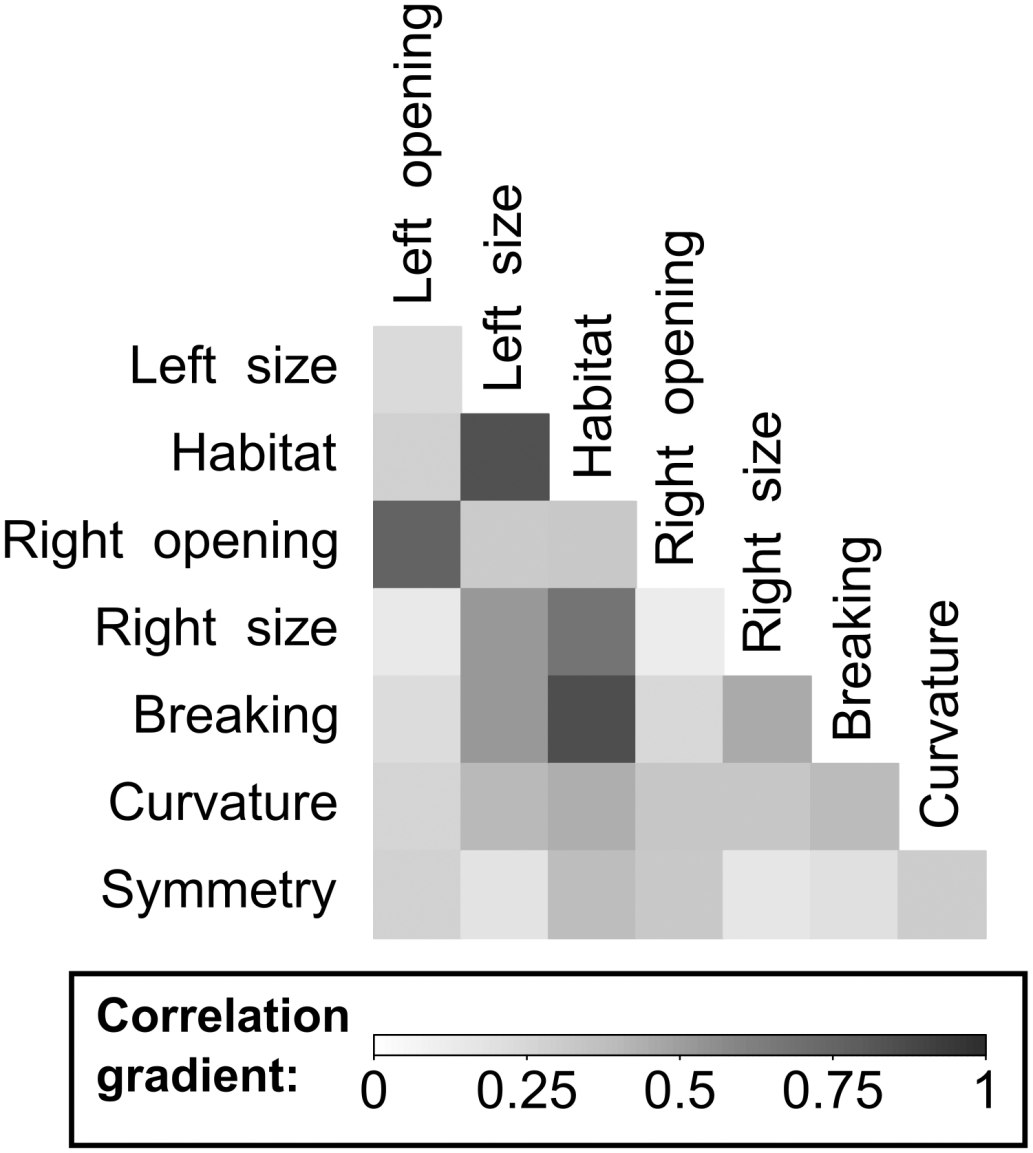
Correlation matrix between the variables of the tusk profile, for Etosha (*N*_Individuals_ = 8) and Kruger specimens (*N*_Individuals_ = 31). The darker the square, the stronger the correlation between the variables.

A correlation matrix was used to show the associations between the variables in the tusk's profiles and habitat (Figure 6). The correlation matrices were carried out using the ‘char_cor’ function, of the ‘creditmodel’ package (Fan, 2022), which calculates the correlation coefficient between variables by Cramer's V. Then, the ‘corrplot’ function of the same package was used to visualise the results. The darker the square, the stronger the correlation between the variables.

### 2.8 Multiple correspondence analyses

**FIGURE 7:**
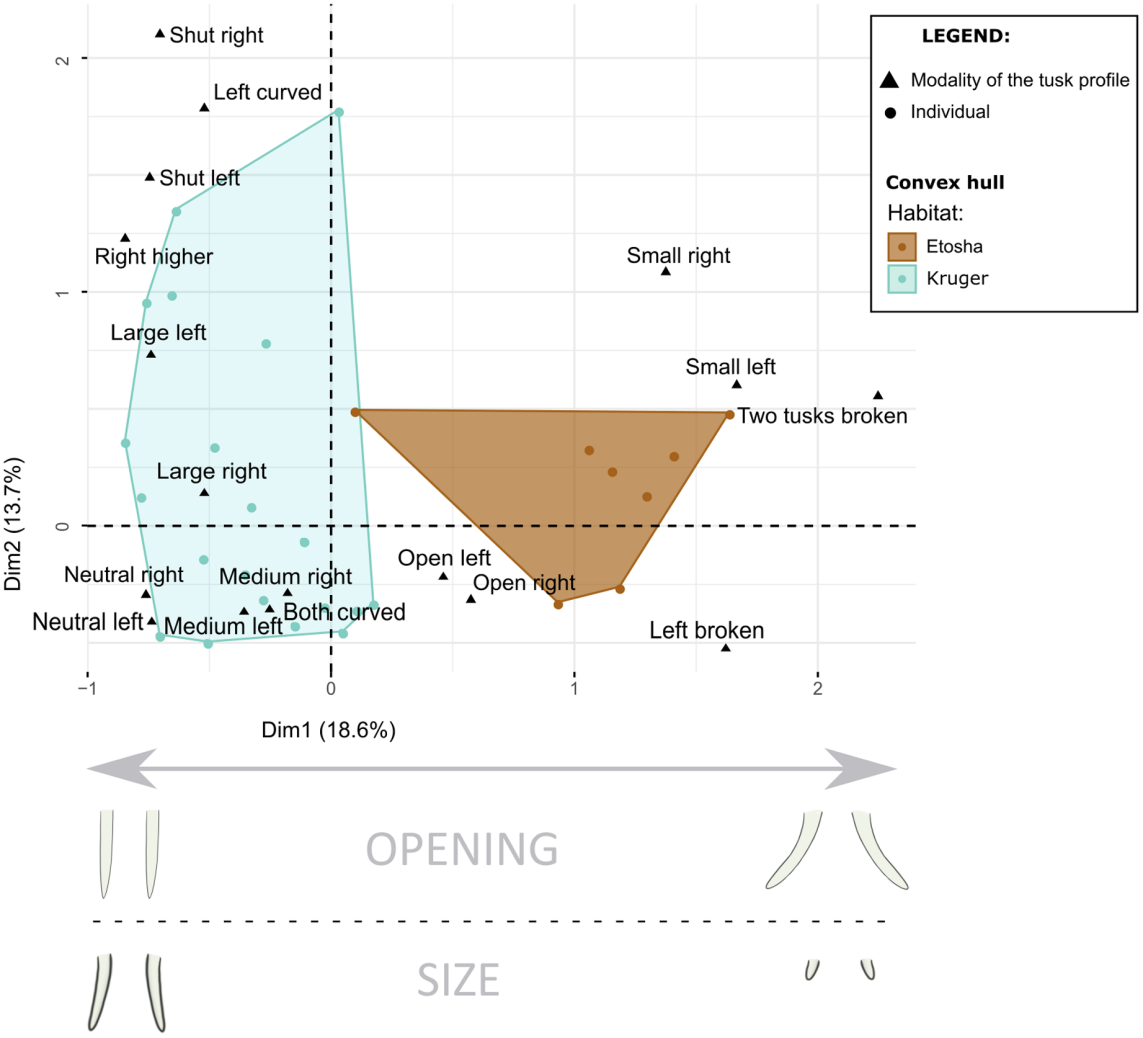
Multiple correspondence analysis plots of the tusk profiles for Etosha (*N*_Individuals_ = 8) and Kruger (*N*_Individuals_ = 31) adult specimens. Dots represent every observed elephant having two tusks and are coloured according to their habitat localisation. Only variables with a contribution greater than or equal to 5% are displayed. Modalities of the tusk profile are displayed in text format.

The MCA was carried out using the ‘MCA’ function of the ‘FactoMineR’ package (Lê et al., 2008). MCA is used to analyse a set of observations described by a set of non‐linear nominal variables and to reduce large sets of variables into smaller sets of components that summarise the information contained in the data. Each nominal variable comprises several modalities (Abdi & Valentin, 2007; Mori et al., 2016). To study the link between the tusk profile and the habitat, the variables entered into the MCA were all the modalities of the tusk profile, i.e. tusk symmetry, presence, curvature, breaking, left and right tusk opening and left and right tusk size, and the additional variables were the habitat and the sex of the elephants. Only the adult elephants, to avoid age‐related bias, presenting two tusks and all tusk profile criteria described have been used for the correlation matrix and MCA. The visualisations (Figure 7) of the first two axes of the MCA were performed using the ‘fviz_mca_biplot’ function of the ‘factoextra’ package (Kassambara & Mundt, 2020). In the biplot, the dots, representing every observed elephant, are coloured according to their habitat. Only variables with a contribution greater than or equal to 5% on the two first axis are displayed.

### 2.9 Permutational multivariate analysis of variance

To test the difference in tusk profile between the selected individuals from both habitats and between sexes a PERMANOVA was performed. As the sex ratio was unbalanced in both habitats, we also checked if there was an interaction between sex and habitat. Thus, a PERMANOVA, a non‐parametric method, was performed on MCA coordinates, using the ‘vegdist’ and ‘adonis2’ functions of the ‘vegan’ package (Oksanen et al., 2022). PERMANOVA is a geometric partitioning of multivariate variation in the space of a chosen dissimilarity measure according to a given ANOVA design, with *p*‐values obtained using appropriate distribution‐free permutation techniques (Anderson, 2017). The test statistic is a multivariate analogue to Fisher's *F*‐ratio calculated directly from any symmetric distance matrix. *p*‐Values were then obtained using 999 permutations (Anderson, 2001).

### 2.10 Fisher's exact test

**FIGURE 8:**
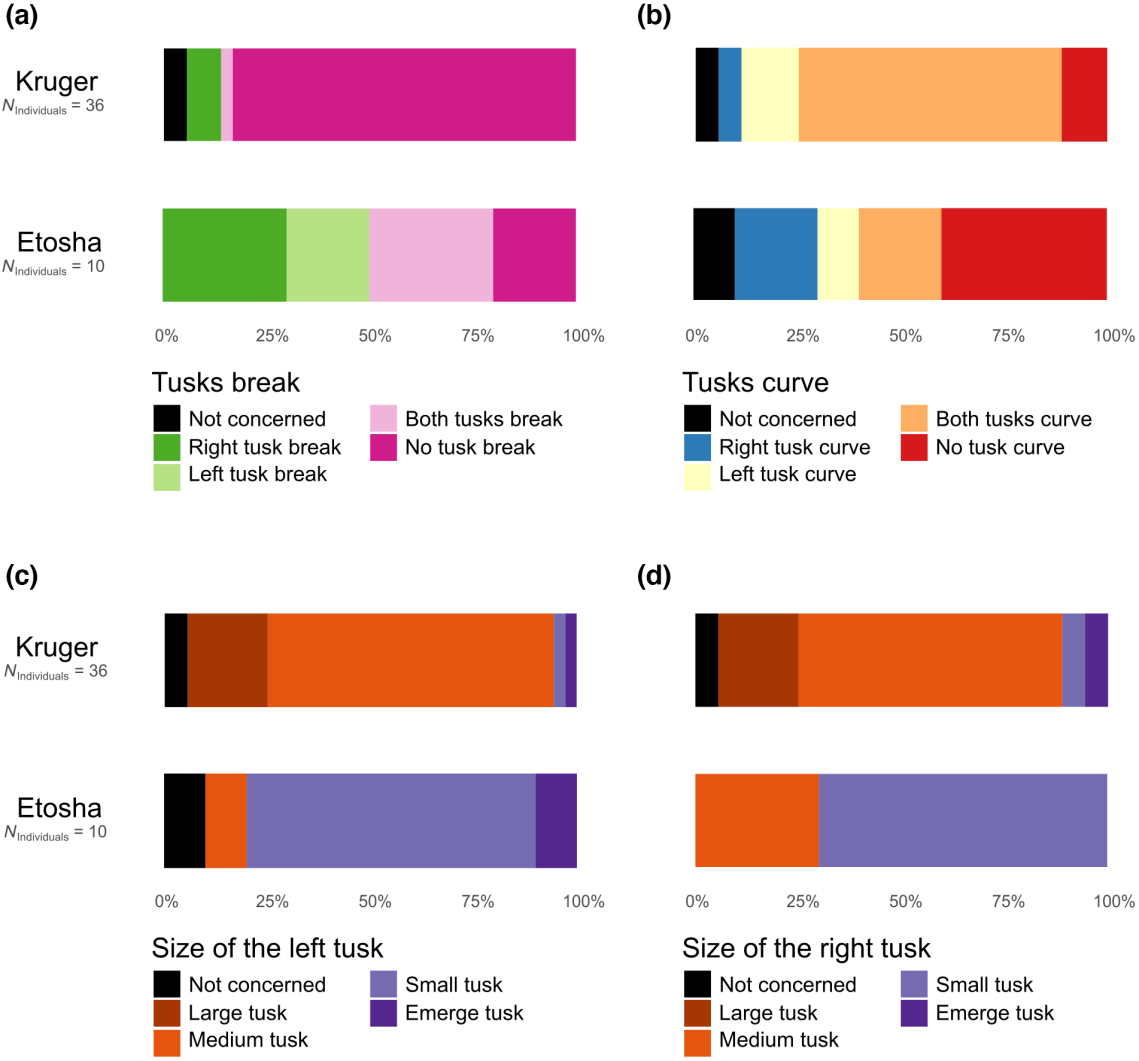
Barplots of adult elephant tusks profiles in the Kruger habitat and Etosha habitat. The different tusk profile criteria significantly different between Kruger and Etosha groups are the tusk break (a), the tusk curvature (b) and the left (c) and right tusk size (d).

Then, to compare the tusk profile of selected adult Etosha and Kruger elephants, the percentages of each of the tusk profile modalities were calculated for each habitat. The distribution of the modalities was then compared between habitats using Fisher's exact test for each criterion of the tusk profile. Elephants not concerned by the criteria were removed for the corresponding Fisher's Exact tests. Fisher's exact test is recommended for the analysis of samples of any size, including small sample sizes and/or imbalanced data, as is the case in our study (De Lima Cabral & De Barros, 2018; Fisher, 1992). We used the ‘fisher.test’ function of the ‘stats’ package (R Core Team, 2021) to carry out the tests. To illustrate the significant differences in distributions of the modalities, bar plots were made on each criterion of the tusks profile (Figure 8). These visualisations were carried out using the ‘ggplot’ function of the ‘ggplot2’ package (Wickham, 2016). For each bar plot, the number of observations (*N*_observations_) and individuals (*N*_individuals_) implicated were reported.

## 3 RESULTS

### 3.1 Trunk grasping techniques according to the habitat and item grasped

The GLMMs show that the individuals have a dissimilar trunk technique for the grasps in Kruger and Etosha. More precisely, the frequency of use of various trunk postures was influenced by the habitat (*χ* ^2^ = 4.084, df = 1, *p* < .05). Both significant and non‐significant GLMM results are summarised in Table S2. Male elephants in Etosha pinched more often and wrapped less often on average than male elephants in Kruger (Figure 3).

The various trunk parts were not used in the same proportions in Etosha and Kruger. There was no statistical difference in the frequency of the use of the base of the trunk between the two habitats during grass grasping (Table S2). On the contrary, the frequency of using only the tip of the trunk while grasping was different between Etosha and Kruger (*χ* ^2^ = 11.605, df = 1, *p* < .001). Using only the tip of the trunk for grasping was more frequent in Etosha than in Kruger (Figure 4).

Elephants in Etosha and Kruger used the same proportion of frontal and lateral grasps to take food items (Table S2). On the contrary, among the lateral grasps, the habitat influenced the grasping direction (*χ* ^2^ = 4.469, df = 1, *p* < .05). Indeed, elephants in Etosha grasped less often from the right and more often from the left on average than elephants in Kruger (Figure 5). Figure 5 also shows that elephants at both Etosha and Kruger mostly grasped from the right.

### 3.2 Differences in tusk profiles in the two habitats and link with the grasping technique

The tusk profiles of the adult Kruger and Etosha elephants were characterised and compared. The results show that most elephants had two tusks (Table S3), and there were no significant differences between Kruger and Etosha habitats concerning the tusk presence (Fisher exact test: *p* = .253; Table S4). Thus, only the elephants presenting two tusks have been used for the MCA (Figure 7; Table S5). The PERMANOVA on the MCA coordinates shows that the habitats had a different variance, that is to say, individuals had a dissimilar tusk profile in Kruger and Etosha (*F* = 11.46, df = 1, *p* = .001; Table S6). There is no sex effect on the tusk profile (Sex: *F* = 1.45, df = 1, *p* = .19). The habitat variable highly correlates to the tusk size and breaking (Figure 6). The habitat variable also showed a correlation with curvature and symmetry variables (Figure 6). The first axis of the MCA allows us to differentiate the two habitats using mostly the tusks' opening and size. Indeed, the Etosha individuals had open, small and broken tusks whereas Kruger individuals had neutral or shut, medium or long and curved tusks (Figure 8).

The comparison of the modality frequencies confirms the existence of differences between the two habitats concerning the tusks breaking and curvature (respectively *p* < .001 and *p* < .05). Kruger elephants had fewer broken and straight tusks than Etosha (Figure 8a,b). Finally, the results also showed that the elephants living in the Kruger habitat had longer tusks than the elephants living in the Etosha habitat (left tusks: Figure 8c, *p* < .001; right tusks: Figure 8d, *p* < .001). The second axis of the MCA highlights two groups of specimens in the Kruger habitat, according to their tusk size, tusk curvature and opening. The shut tusks were associated with the left curved tusks and long tusks whereas the neutral tusks were related to both curved tusks and the medium size (Figure 6). However, the tusk opening and symmetry also seemed to be similar between the habitats (Left tusk opening: *p* = .378; right tusk opening: *p* = .197; tusk symmetry: *p* = .145).

To understand links between the tusks profile and the trunk grasping technique we compared the frequency of the use of the variables' modalities of the grasping technique according to the tusks' profile.

**FIGURE 9:**
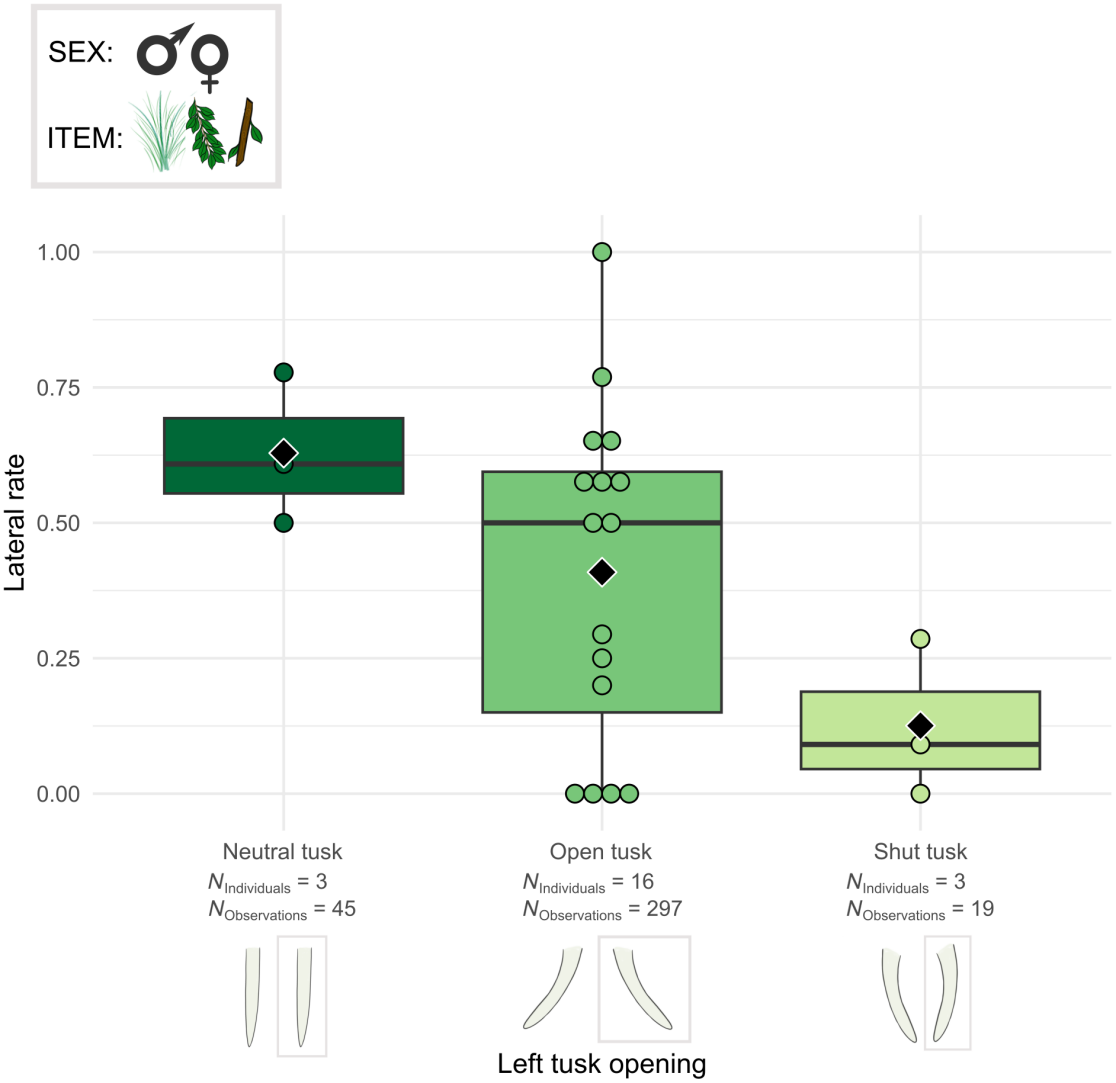
Boxplots of the lateral grasp rate per male and female individual according to the left tusk opening profile. The grasped food items were the grass, leaves and small branches. Individuals from Kruger and Etosha were all considered. Black diamonds indicate the average lateral rate for each left tusk opening profile. Chi‐squared test: *p* < .05.

The grasping direction (lateral vs. frontal) was related to the left tusk opening (*χ* ^2^ = 8.032, df = 2, *p* < .05). Elephants with a left neutral tusk grasped more from the side than elephants having an open or shut left tusk and elephants with a left shut tusk grasped more frontally than elephants having an open or neutral left tusk (Figure 9). On the contrary, other modalities of the trunk grasping technique were not related to the left tusk opening (Table S2).

**FIGURE 10:**
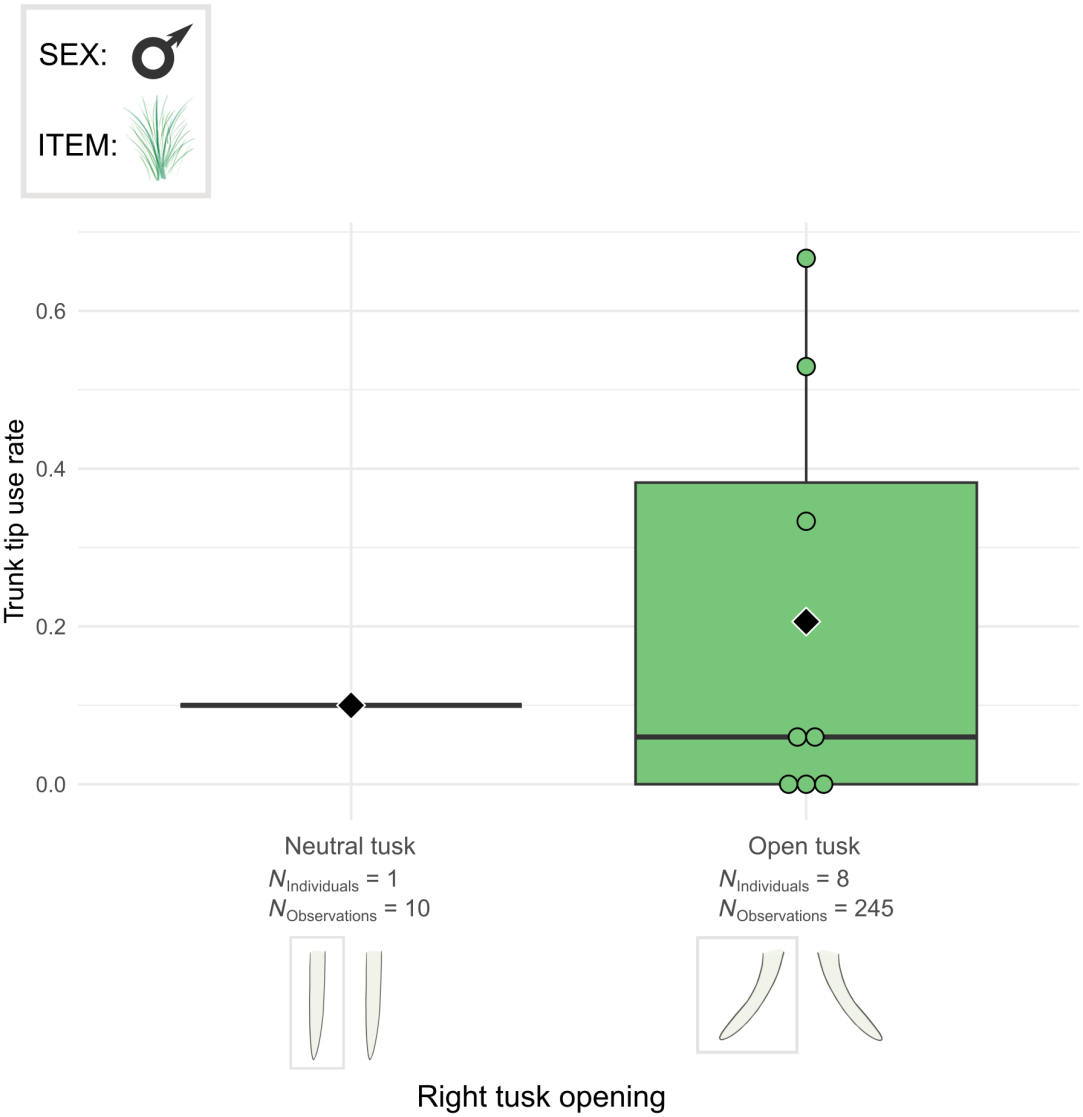
Boxplots of the tip grasp rate per male individual according to the right tusk opening profile. The grasped food item was the grass. Individuals from Kruger and Etosha were all considered. Black diamonds indicate the average trunk tip use rate for each right tusk opening profile. Chi‐squared test: *p* < .05.

The frequency of using only the tip of the trunk while grasping was related to the right tusk opening (*χ* ^2^ = 5.282, df = 1, *p* < .05). Elephants with a right neutral tusk grasped with only the trunk tip less often than elephants having an open right tusk (Figure 10). On the contrary, other modalities of the trunk grasping technique were not related to the right tusk opening (Table S2).

**FIGURE 11:**
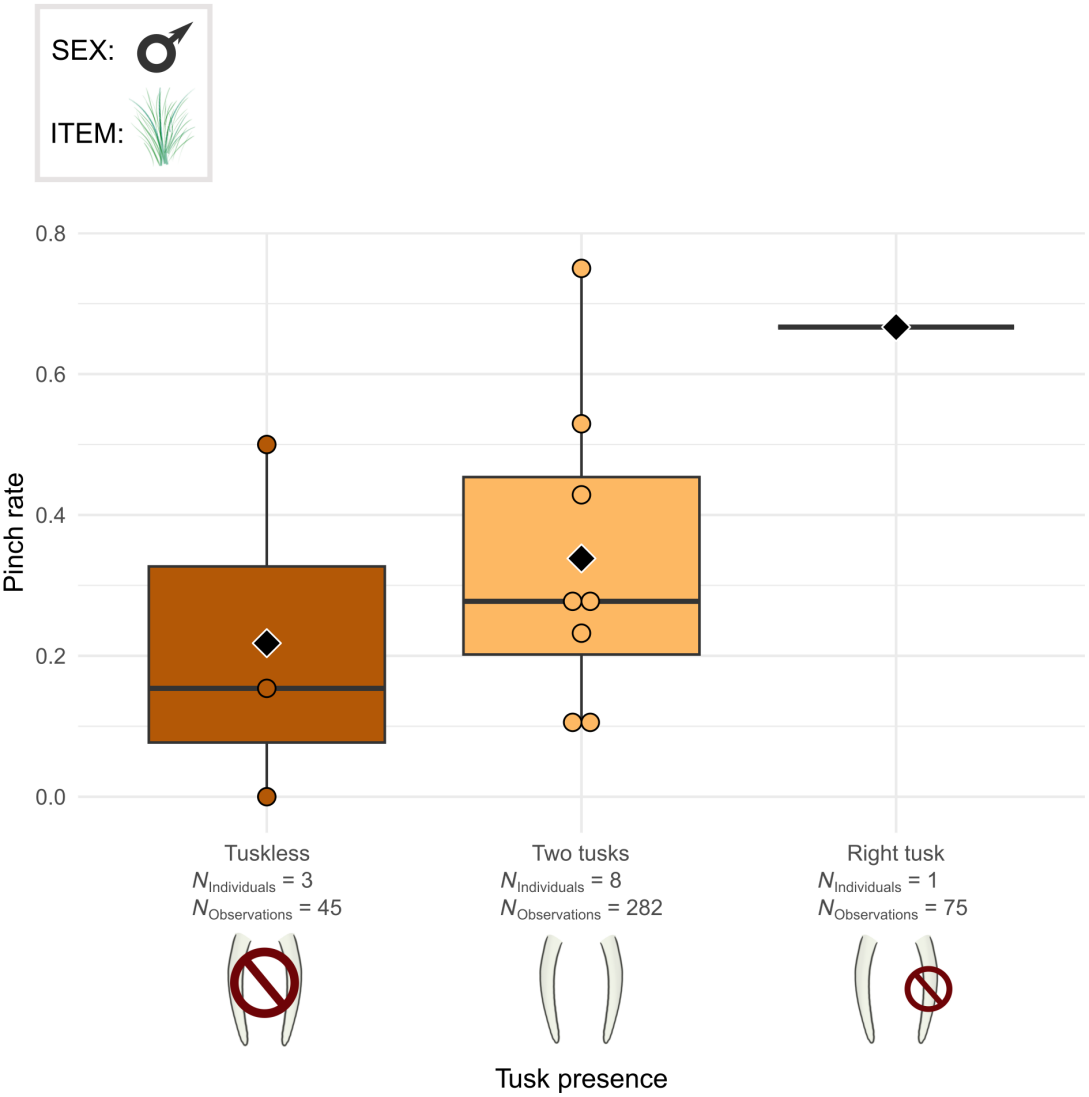
Boxplots of the pinch rate per male individual according to the tusk presence profile. The grasped food items were grass, leaves and small branches. Individuals from Kruger and Etosha were all considered. Black diamonds indicate the average pinch rate for each tusk‐presence profile. Chi‐squared test: *p* < .001.

**FIGURE 12:**
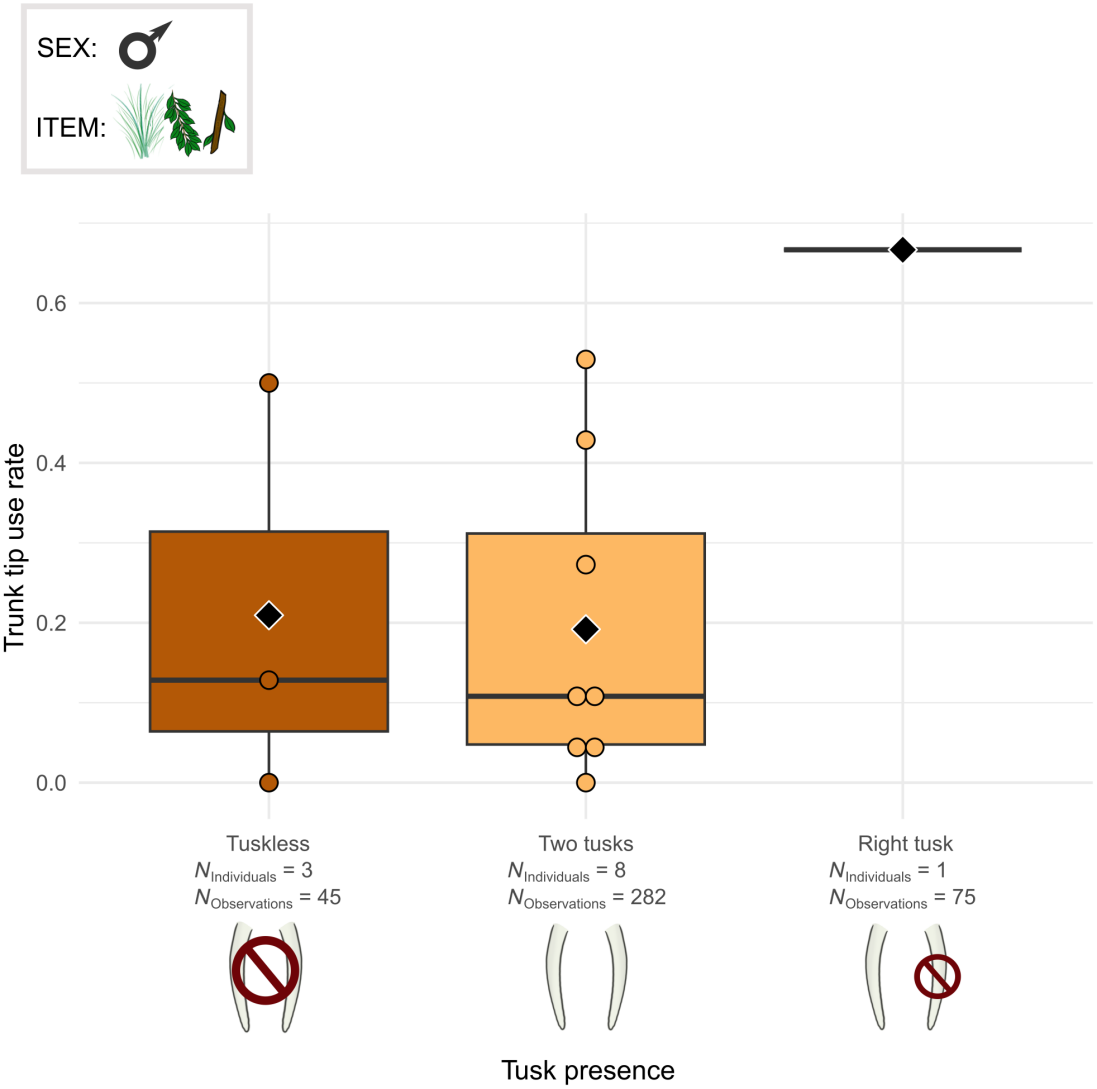
Boxplots of the tip grasp rate per male individual according to the tusk presence profile. The grasped food items were grass, leaves and small branches. Individuals from Kruger and Etosha were all considered. Black diamonds indicate the average trunk tip use rate for each tusk‐presence profile. Chi‐squared test: *p* < .05.

The tusk presence was also related to several modalities of the trunk grasping technique. The trunk posture (*χ* ^2^ = 18.966, df = 2, *p* < .001) and the use of the tip of the trunk were linked with the tusk presence in male elephants (*χ* ^2^ = 6.718, df = 2, *p* < .05). In males, the tuskless individuals pinched less often than the elephants with two tusks (Figure 11), whereas their tip use rates were quite similar (Figure 12). The elephant with only the right tusk pinched more (Figure 11) and used only the trunk tip more often on average than all other elephants (Figure 12).

The conditions for applying the models with curvature data were not met, so the models could not be used. The same issue applies to models testing the relationships between trunk posture and left tusk size, as well as the trunk posture, grasping direction (right vs. left), use of the trunk base and right tusk size. Excluding the models that did not make it, no modality of the trunk grasping technique was related to the left and right tusk's size, as well as to the tusk symmetry (Table S2).

**FIGURE 13:**
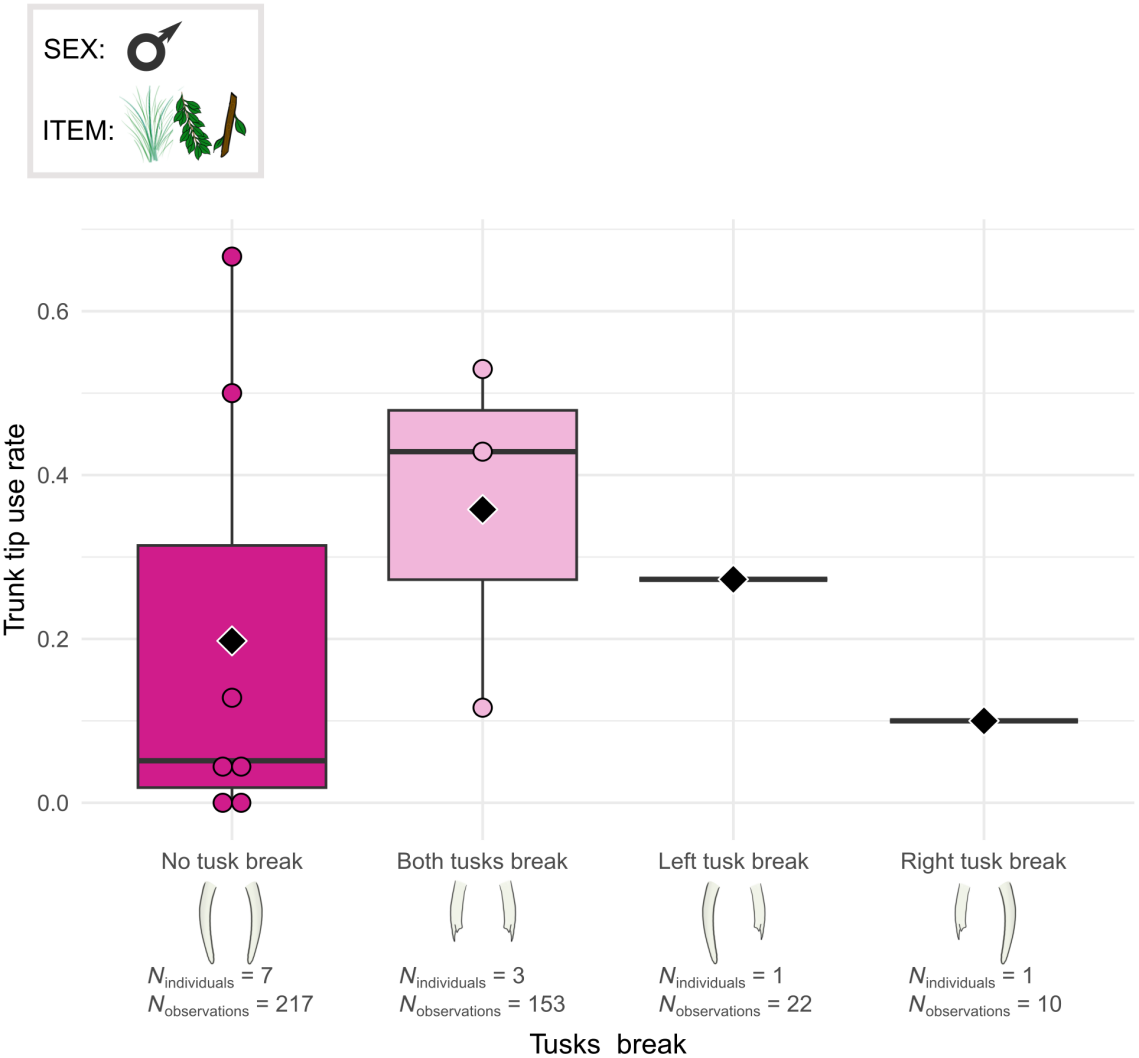
Boxplots of the tip grasp rate per male individual according to the tusk‐breaking profile. The grasped food items were grass, leaves and small branches. Individuals from Kruger and Etosha were all considered. Black diamonds indicate the average trunk tip use rate for each tusk‐breaking profile. Chi‐squared test: *p* < .01.

Any modality of the trunk grasping technique was related to the tusk‐breaking profile, except the frequency of the specific use of the trunk tip (*χ* ^2^ = 15.076, df = 3, *p* < .01, Table S2). In males, the individuals with two intact tusks used only the tip of the trunk to grasp food items less often than individuals with both tusks broken. The individual with only the left tusk broken had an intermediate rate of trunk tip use whereas the individual with only the right tusk broken had a lower rate of trunk tip use compared to the others (Figure 13).

## 4 DISCUSSION

### 4.1 Trunk grasping techniques according to habitat

Our results revealed that the use of trunk grasping techniques differed between the two studied groups of elephants living in two different habitats (i.e. Kruger and Etosha).

Consistent with our prediction, we revealed that the technique's modalities used for grasping grass, leaves and small branches differed between the studied specimens of the two habitats. Compared to Kruger male elephants, Etosha male elephants used the pinch technique more, thus recruiting only the tip of the trunk more. We described above that, even if all studied sites had the same vegetation type, i.e. mopane tree and grass, the elephant groups faced different food accessibility (Codron et al., 2006; Estes, 1999; Sach et al., 2019). Indeed, the studied sites in Etosha National Park are drier habitat, with less and lower grass at the disposal of the elephants than in the Kruger National Park sites (Kennedy et al., 2003; Sannier et al., 2002). Moreover, despite their ability to compose with water stress conditions, mopane trees and bushes' size and architecture are dependent on the rainfall, with smaller growth in dry habitats (O'Connor, 1998) and a reduction of leaves availability and quality (Makhado et al., 2018; Stevens et al., 2016). Thus, in Etosha, the small branches and leaves are in a lower position compared to the ones in Kruger, and the leaves are less at disposition to the elephant and likely more friable. It has been previously reported, in ex‐situ experiments, that the use of trunk grasping techniques is related to the quantity, shape and size of the item grasped (Dagenais et al., 2021; Lefeuvre et al., 2020; Racine, 1980) and the distal trunk morphology (Soppelsa et al., 2022). Grass, small branches and leaves, being scarcer resources in Etosha, and lower in size, might require a more parsimonious collection and thus the use of the pinch, which is more precise than the wrap (Rasmussen & Munger, 1996). Furthermore, as the mopane leaves tend to fold together under high temperatures like in Etosha (Makhado et al., 2014), it could be more practical for the Etosha elephant to grasp with the trunk tip these folded leaves. On the contrary, in Kruger sites, elephants used more wraps to grasp grass, leaves and small branches. From our observation and the literature on the Etosha and Kruger vegetation, we assume that the patches of grass and the mopane trees and bushes were denser and higher in Kruger than in Etosha, allowing the elephants in Kruger to use more the wrapping technique. Moreover, the firmness of the tie between the item and its substrate may have an impact on the force required to extract specific food items, and thus how the trunk is used. This tie is particularly related to the aridity of the habitat, as the ground moisture may impact its grass attachment, and the small branch and leaves can be more fragile in drier environments. The age of the elephant may therefore also impact the use of the trunk related to the habitat, as young elephants will most likely not have the same strength of grown adult elephant to detach these food items. One limitation of our study is that we were unable to directly characterise or quantify the vegetation on the sites where the study was carried out, although our field observations were consistent with the information found in the literature.

On the other hand, we highlighted that, despite a global right bias in prehension, i.e. a functional asymmetry in the use of the trunk to grasp food, which bends or wraps more to the right than to the left from the elephant's point of view, consistent with the literature (Giljov et al., 2017, 2018; Keerthipriya et al., 2015; Lefeuvre et al., 2022; Revathe et al., 2020) and our prediction, trunk direction bias differs between the two habitats when grasping food items. Even if there is a similar proportion of frontal and lateral grasps in the two habitats, the Kruger elephants used more right grasps than the Etosha elephants. Indeed, we assumed previously that the Kruger elephants use more wraps due to the food properties in their habitats, yet the wraps require complex trunk trajectories (Dagenais et al., 2021). According to the ‘task complexity theory’ valorised in primates (Fagot & Vauclair, 1991), the right biases are more important in Kruger elephants due to the complexity of their food grasps in this habitat.

Between groups, variations in grasping techniques could also result from differences in social learning and transmission. Technique transmission has been documented in several species, e.g. in pigeons and vervet monkeys (Canteloup et al., 2020; Palameta & Lefebvre, 1985). This social learning can lead to differences in techniques between groups of wild animals of the same species (Humle & Matsuzawa, 2001; Ottoni & Izar, 2008). Therefore, as elephants are social beings, trunk techniques could be the outcome of transmission between individuals, especially mothers and allomothers, to calves.

### 4.2 Differences in tusks profiles according to the habitat

Differences in the size of the tusks, the absence of one or two tusks, and even breaking of the tusks have been investigated in different African savannah elephant groups (Bielert et al., 2018; Campbell‐Staton et al., 2021; Chiyo et al., 2015; Elder, 1970; Layser & Buss, 1985), including elephants from the Kruger National Park and Etosha National Park (Steenkamp et al., 2007; Whyte & Hall‐Martin, 2018).

We observed no significant difference in terms of tusk presence between elephants living in the Kruger versus Etosha habitat, which matches previous findings from Steenkamp et al. (2007). We found a tusklessness, i.e. the absence of both tusks, below 5% for the Kruger elephants just like Raubenheimer stated in 2000. We did not observe any elephant from Etosha habitat with tusklessness, similar to Steenkamp et al. (2007). Yet, it was revealed that the tusklessness in elephant groups is directly related to their poaching history (Campbell‐Staton et al., 2021; Chiyo et al., 2015; Heimert, 1994). Thus, we can presume, in agreement with Whyte and Hall‐Martin (2018), that the low poaching in both habitats due to their protected status (national parks) can explain the low frequencies of tusklessness, contrary to other elephant groups that experience strong poaching (Campbell‐Staton et al., 2021).

We found a significant difference in terms of breaking of the tusks between the two groups investigated, which is in continuation with the results reported 16 years ago by Steenkamp et al. (2007). In both habitats (i.e., Etosha and Kruger), we found higher percentages of broken tusks than reported in the previous study. Regarding Etosha elephants, Steenkamp et al. (2007) reported 44.4% of broken tusks while 73% of the elephants we observed had one or both tusks break. In Kruger's elephants, these authors observed only 1.64% of tusks break while we observed nearly 2% of the elephants with one or both tusks break. This difference may be due to the difference in sample sizes between the two studies. Nevertheless, the strong tusk break difference between the two habitats seems steady over time. This significant difference in the percentage of breaking tusk between the two groups may be explained by environmental differences between their respective habitats. Fluoride is a chemical that is abundant in Etosha's waterholes, probably due to low rainfall (Auer, 1997). Ingestion of high levels of this chemical by elephants could cause crumbly tusks and thus a higher chance of breaking, thus explaining the higher tusk break incidence in elephants from Etosha (Raubenheimer, 1999; Raubenheimer et al., 1998; Steenkamp et al., 2007). Moreover, it has been previously documented that elephants also use their tusks for digging (Steenkamp, 2003). Differences in the soil composition between the two habitats might be another factor explaining the different percentages of tusk break between the two groups. A more abrasive or gravelly soil would lead to shorter and more breakable tusks. Also, differences in the elephants' diet might be a factor to consider as a mineral salt deficiency could induce more friable tusks. Exploring precisely which elements of their diet may impact the development of the tusks and frailty could be of interest to further understanding variability in the tusk profile between elephant groups.

In addition, we found that the tusks' sizes, which may be linked to possible break or their development, differ between the elephants of Kruger and Etosha habitats, as well as their tusk curvatures. The right and left tusks displayed similar patterns of size per habitat, but the tusks of Etosha elephants were significantly smaller, 80%–87% of the tusks (right and left respectively) are emergent or small in Etosha versus 27%–20% in Kruger, certainly due to the high friability and break incidence explain above (Whyte & Hall‐Martin, 2018). The tusks of the Etosha elephant were also less curved than those of the Kruger elephants. A link between the size of the tusk and its curvature could exist: the longer, the more curved the tusks are. Indeed, with a longer size and fewer breaking (Whyte & Hall‐Martin, 2018), the tusks of Kruger's elephants displayed a greater curvature. It can be interesting for further studied to explore the link between the size and the curvature of the tusks. We did not find any difference in tusk symmetry and opening between the two habitats.

### 4.3 Tusk profiles and trunk grasping technique relationship

Differences in the use of trunk grasping techniques were partly associated with the variations we observed in the different tusk features. Elephants with their right tusk open used more the trunk tip than the elephant with neutral tusk, certainly due to individual preference as there is only one individual with neutral tusks. Interestingly, the use frequency of frontal and lateral grasps differed with the opening of the left tusks. Elephants with their left tusk shut used more frontal grasps, whereas elephants with their left tusk neutral will use more lateral grasps. Elephants with open left tusks made almost as many lateral grasps as frontal ones, with a slight predominance of frontal grasps. These results suggest that a rotation of the left tusk towards or away from the trunk may interfere with lateral grasping movements of the trunk. On the contrary, a more open left tusk probably enables the trunk more freedom of movement, and thus the use of both frontal and lateral grasps. The left tusk is particularly involved in this trunk obstruction as the studied elephants, when side grasp, grasp predominantly to the right, and therefore the base of the trunk shifts to the left. Precisely, in a right trunk grasp, a shut left tusk is more likely to impede the left movement of the base of the trunk. However, as we previously established that the elephants in Etosha and Kruger used the same proportion of frontal and lateral grasps, this difference in trunk technique related to the tusk profiles is not related to the habitats.

Moreover, there is differences in the use of the pinch and in the use of the trunk tip only in relation with the tusk presence. First, the tuskless male elephants used the pinch less than the elephants that have their both tusks. We hypothesised that the presence of tusks could obstruct the wrapping process, and thus the elephants with tusks may favour the use of the pinch instead of the wrap. The tusk presence may therefore have an impact on the trunk techniques, but this is not correlated to the habitat, as we did not find any difference in tusk presence between the Etosha and Kruger elephants. With the evolution of the tusks towards tusklessness due to poaching pressure in numerous elephant groups (Campbell‐Staton et al., 2021; Chiyo et al., 2015; Heimert, 1994), African savannah elephants thus could use in the future the wrap more. Besides, one male with only a right tusk used the pinch and the trunk tip only more than the elephants that have both tusks or none. This result highlight that the individual preferences in terms of trunk techniques are also important in the studied elephants.

Finally, the breaking of the tusks had an impact on the trunk grasping techniques between the two habitats. Male elephants with both intact tusks used slightly less the trunk tip only than male elephants having both tusks broken. The male with one broken tusk had a higher tip use rate than the other two tusk profiles. We previously stated that there are more break tusks in Etosha than in Kruger, probably related to the habitat, and we also previously demonstrated that Etosha elephants use the trunk tip more than Kruger elephants, about the food items accessibility. Therefore we can presume that these elephants with broken tusks that use the trunk tip more are the Etosha elephants. Drier habitats may influence elephant tusk breakability as well as elephant trunk techniques. With the aridification of a majority of elephant habitats due to climate change, we can imagine that both tusk friability and trunk techniques may change in African savannah elephants in the future.

The study also highlighted the difficulty of identifying young elephants. Infants and juveniles often do not yet have scars on their bodies or broken tusks. This difficulty explains why we have relatively few specimens in these age classes. To improve this ability to identify elephants, the creation of a public database listing the identification of all known elephants within the Kruger and Etosha parks would be of great use. This concept has already been implemented and proven effective for the populations of Maasai Mara in Kenya and Gorongosa National Park in Mozambique (‘Who's Who & Whereabouts’ database; Granli & Poole, 2022).

In conclusion, trunk grasping techniques, such as the trunk posture and the grasping direction bias, differ between different elephant groups in relation to their habitats, and in particular the density and size of the vegetation. The individual preferences and social learning may also have an incidence on these grasping techniques. Concerning the tusks, their breaking, size and curvature, can also differ between different elephant groups and in relation to their habitats too. Indeed, the minerals, fluoride levels and soil composition may have an impact on the tusk breakability, and they differ between the studied habitats. Finally, the tusk presence, and particularly the ones facing the trunk, may interfere with lateral and wrap grasping movements but not in relation to the habitat. Moreover, the habitat have an impact on both trunk posture and tusk breakability. This study was based on a sample of 64 wild African savannah elephants from two habitats, with both sexes, diverse age classes and diverse tusk profiles. To confirm these results, it would be interesting to increase the sample size with more similar tusk profile, sex and age class elephants.

## AUTHOR CONTRIBUTIONS

**Pauline Costes:** Data curation (equal); formal analysis (equal); visualization (equal); writing – original draft (equal); writing – review and editing (equal). **Julie Soppelsa:** Data curation (equal); formal analysis (equal); resources (equal); visualization (equal); writing – original draft (equal); writing – review and editing (equal). **Céline Houssin:** Methodology (equal); resources (equal); writing – review and editing (equal). **Grégoire Boulinguez‐Ambroise:** Resources (equal); writing – review and editing (equal). **Camille Pacou:** Resources (equal); writing – review and editing (equal). **Patrick Gouat:** Methodology (equal); writing – review and editing (equal). **Raphaël Cornette:** Conceptualization (equal); methodology (equal); resources (equal); writing – review and editing (equal). **Emmanuelle Pouydebat:** Conceptualization (equal); funding acquisition (equal); methodology (equal); writing – review and editing (equal).

## CONFLICT OF INTEREST STATEMENT

The authors declare that there are no conflicts of interest.

## Supporting information


Data S1

Figure S1

Tables S1–S5

Table S6

## Data Availability

The data that supports the findings of this study are available in the Supporting Information of this article.
