# Supplementary figures and images for: Effect of the habitat and tusks on trunk grasping techniques in African savannah elephants
Source: Ecol Evol. 2024 Apr 19;14(4):e11317. doi: 10.1002/ece3.11317 (PMC11027014; doi:10.1002/ece3.11317)

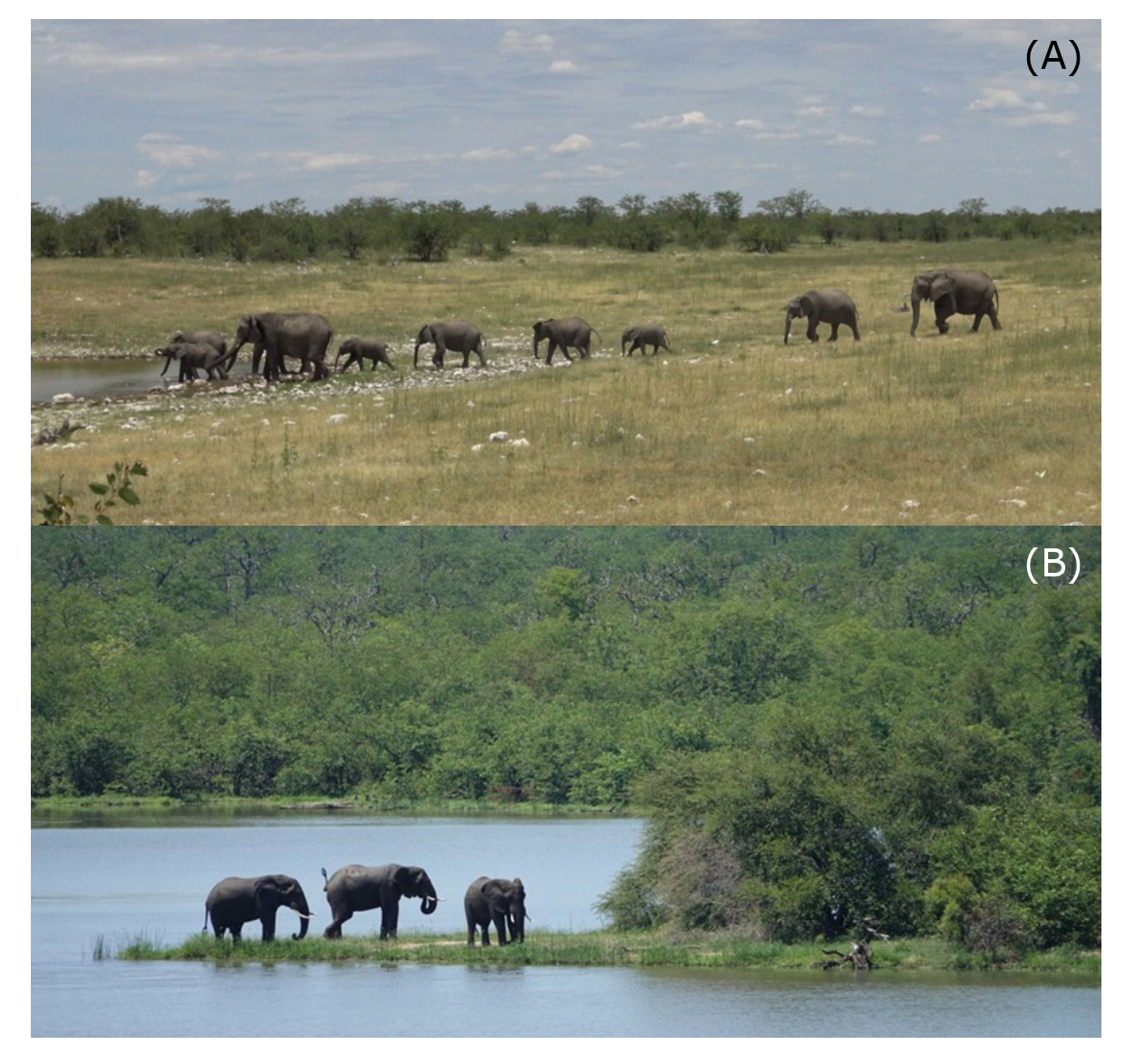

Supplement: Supplementary file 2 — Figure S1 [file ECE3-14-e11317-s004.tif]
