# Supplementary material for: Effect of the habitat and tusks on trunk grasping techniques in African savannah elephants
Source: Ecol Evol. 2024 Apr 19;14(4):e11317. doi: 10.1002/ece3.11317 (PMC11027014; doi:10.1002/ece3.11317)
Supplement: Supplementary file 3 — Tables S1–S5 [file ECE3-14-e11317-s002.docx]

**Supplementary material**

**Figure S1** – Illustration of the different study sites: (A) Aus in Etosha Park and (B) Pioneer Dam in Kruger Park. Pictures by J. Soppelsa.

**Table S1** – Number of food item grasp observed for each elephant per habitat.

| **Habitat** | **Individual ID** | **Observations (n=)** |
| --- | --- | --- |
| Etosha | fa03 | 3 |
|  | fa06 | 3 |
|  | fs01 | 1 |
|  | ma01 | 10 |
|  | ma02 | 34 |
|  | ma03 | 75 |
|  | ml01 | 7 |
|  | ml02 | 22 |
|  | s02 | 2 |
|  | i01 | 1 |
|  | i02 | 2 |
|  | i04 | 4 |
| Kruger | Fa101 | 1 |
|  | Fa104 | 11 |
|  | Fa105 | 1 |
|  | Fa113 | 4 |
|  | Fa2 | 5 |
|  | Fa3 | 1 |
|  | Fa7 | 27 |
|  | Fa8 | 7 |
|  | FaX | 23 |
|  | Fs1 | 7 |
|  | Fs101 | 1 |
|  | Fs2 | 1 |
|  | Fj1 | 2 |
|  | Fj101 | 4 |
|  | Ma101 | 4 |
|  | Ma106 | 112 |
|  | Ms102 | 54 |
|  | Ms4 | 39 |
|  | Mj2 | 39 |
|  | j1 | 6 |
|  | Mi101 | 4 |
|  | Mi102 | 2 |

**Table S2** – Sample size for each modality of the tusk profile per habitat.

| **Tusks profile variables** | **Modalities** | **Individuals (N=)** | |
| --- | --- | --- | --- |
|  |  | **Etosha** | **Kruger** |
| Presence | Tuskless | 0 | 2 |
|  | Both tusks | 14 | 44 |
|  | Right tusk | 1 | 0 |
| Breaking | No breaking | 4 | 40 |
|  | Both tusks broken | 5 | 1 |
|  | Left tusk broken | 3 | 0 |
|  | Right tusk broken | 3 | 3 |
|  | Not concerned | 0 | 2 |
| Curvature | Both tusks straight | 9 | 8 |
|  | Both tusks curved | 2 | 27 |
|  | Left tusk curved | 1 | 7 |
|  | Right tusk curved | 2 | 2 |
|  | Not concerned | 1 | 2 |
| Symmetry | Symmetric tusks | 13 | 27 |
|  | Right higher | 0 | 4 |
|  | Left higher | 0 | 10 |
|  | Not concerned | 2 | 5 |
| Left size | Emergent | 1 | 1 |
|  | Small | 12 | 8 |
|  | Medium | 1 | 28 |
|  | Large | 0 | 7 |
|  | Not concerned | 1 | 2 |
| Right size | Emergent | 0 | 2 |
|  | Small | 12 | 10 |
|  | Medium | 3 | 25 |
|  | Large | 0 | 7 |
|  | Not concerned | 0 | 2 |
| Left opening | Open | 11 | 27 |
|  | Neutral | 2 | 8 |
|  | Shut | 0 | 8 |
|  | Not concerned | 2 | 3 |
| Right opening | Open | 11 | 23 |
|  | Neutral | 2 | 13 |
|  | Shut | 0 | 7 |
|  | Not concerned | 2 | 3 |

**Table S3** – Contributions of the variables, displayed in the ACM (Figure 8), greater than or equal to 5% on axes 1 and 2.

|  | **Contributions (%)** | | |
| --- | --- | --- | --- |
| **Modality** | **Axis 1** | **Axis 2** | **Sum** |
| No tusk broken | 4.05 | 0.07 | 4.12 |
| **Two tusks broken** | **8.7** | **0.72** | **9.42** |
| **Left tusk broken** | **4.53** | **0.65** | **5.18** |
| Right tusk broken | 4.06 | 0.48 | 4.54 |
| Both straight tusks | 3.21 | 0.95 | 4.16 |
| **Both curved tusks** | **1.36** | **3.75** | **5.11** |
| **Left curved tusk** | **0.93** | **14.93** | **15.86** |
| Right curved tusk | 2.84 | 0.05 | 2.89 |
| Symmetrical tusks | 2.69 | 0.88 | 3.57 |
| Left tusk higher | 2.56 | 0.04 | 2.6 |
| **Right tusk higher** | **2.46** | **7.06** | **9.52** |
| **Small left** | **19.14** | **3.39** | **22.53** |
| **Medium left** | **2.73** | **3.97** | **6.7** |
| **Large left** | **2.82** | **3.76** | **6.58** |
| **Small right** | **9.77** | **8.27** | **18.04** |
| Medium right | 0.71 | 2.53 | 3.24 |
| Large right | 1.63 | 0.16 | 1.79 |
| **Neutral left** | **4.19** | **1.77** | **5.96** |
| **Open left** | **4.41** | **1.34** | **5.75** |
| **Shut left** | **2.87** | **15.58** | **18.45** |
| **Neutral right** | **5.98** | **1.23** | **7.21** |
| **Open right** | **6.25** | **2.58** | **8.83** |
| **Shut right** | **2.13** | **25.87** | **28** |

**Table S4** – Results of the PERMANOVA of the trunk technique and tusk profile between Etosha and Kruger habitats based on Euclidean distances. *Pseudo-F* statistics were calculated for each term using direct analogues to univariate expectations of mean squares. P were obtained using 999 raw data permutations.

|  |  | **Df** | **SumOfSqs** | **R2** | **F** | **P** |
| --- | --- | --- | --- | --- | --- | --- |
| **Tusk profile** | Habitat | 1 | 13.072 | 0.23350 | 11.4564 | 0.001 |
|  | Sex | 1 | 1.656 | 0.02957 | 1.4510 | 0.184 |
|  | Habitat: Sex | 1 | 1.321 | 0.02359 | 1.1573 | 0.341 |

**Table** **S5** – P-value of the Fisher's Exact Test on proportions of the tusk profile modalities:

|  | P |
| --- | --- |
| Tusk presence | 0.253 |
| **Tusk breaking** | **0.0001832** |
| **Tusk curve** | **0.02807** |
| Tusk symmetry | 0.1454 |
| Left opening | 0.3776 |
| Right opening | 0.197 |
| **Left size** | **7.758e-06** |
| **Right size** | **0.0006803** |
