# Supplementary material for: Effect of the habitat and tusks on trunk grasping techniques in African savannah elephants
Source: Ecol Evol. 2024 Apr 19;14(4):e11317. doi: 10.1002/ece3.11317 (PMC11027014; doi:10.1002/ece3.11317)
Supplement: Supplementary file 4 — Table S6 [file ECE3-14-e11317-s001.docx]

**Table S6** - Summary of GLMM data and results

| **Response variable**  **(binary)** | **Sex studied** | **Random effect** | **Fixed effect** | **# Individuals per category** | **# Observations per category** | **Chisq** | **Df** | **P-value** | **Figure** |
| --- | --- | --- | --- | --- | --- | --- | --- | --- | --- |
| *Trunk grasping techniques according to habitat and item grasped* | | | | | | | | | |
| Trunk posture: pinch (1) vs wrap (0) | Males | Individuals, age class | Park*Item | Etosha: 5  Kruger: 7  Small branch: 3  Leaves: 4  Grass: 12 | Etosha: 148  Kruger: 254  Small branch: 33  Leaves: 69  Grass: 300 | Park: 4.0835  Item: 1.6494    Park:Item: 0.4549 | Park: 1  Item: 2    Park:Item: 2 | Park: 0.0433 *  Item: 0.4384    Park:Item: 0.7966 | Fig 3 |
| Grasping direction RL: right (1) vs left (0) | Both | Individuals, age class, Sex | Park*Item | Etosha: 8  Kruger: 15  Small branch: 5  Leaves: 4  Grass: 20 | Etosha: 58  Kruger: 206  Small branch: 23  Leaves: 40  Grass: 201 | Park: 4.4690  Item: 8.0063    Park:Item: 3.2597 | Park: 1  Item: 2    Park:Item: 2 | Park: 0.03452 *  Item: 0.01826 *    Park:Item: 0.19596 | Fig 4  Fig 5 |
| Grasping direction LF: lateral (1) vs frontal (0) | Both | Individuals, age class, sex | Park*Item | Etosha: 12  Kruger: 22  Small branch: 8  Leaves: 6  Grass: 30 | Etosha: 159  Kruger: 355  Small branch: 39  Leaves: 73  Grass: 402 | Park: 1.3131  Item: 1.3014    Park:Item: 0.4356 | Park: 1  Item: 2    Park:Item: 2 | Park: 0.2518  Item: 0.5217    Park:Item: 0.8043 | X |
| Trunk base use: yes (1) vs no (0) | Both | Individuals, age class, sex | Park | Etosha: 9  Kruger: 21 | Etosha: 146  Kruger: 261 | Park: 0 | Park: 1 | Park: 0.9997 | X |
| Trunk tip use only: yes (1) vs no (0) | Both | Individuals, age class, sex | Park*Item | Etosha: 12  Kruger: 22  Small branch: 8  Leaves: 6  Grass: 30 | Etosha: 164  Kruger: 355  Small branch: 39  Leaves: 73  Grass: 407 | Park: 11.6048  Item: 9.4137    Park:Item: 4.6860 | Park: 1  Item: 2    Park:Item: 2 | Park: 0.0006578 ***  Item: 0.0090330 **    Park:Item: 0.0960381 | Fig 6  Fig 7 |
| *Trunk grasping techniques according to the tusk profile* | | | | | | | | | |
| Trunk posture: pinch (1) vs wrap (0) | Both | Individuals, age class, sex, park | Tusk breaking*Item | Right tusk break: 3  Left tusk break: 2  Both tusks break: 4  No tusk break: 22 | Right tusk break: 17  Left tusk break: 23  Both tusks break: 156  No tusk break: 316 | Tusk break: 1.2774  Item: 1.0565  Tuskbreak:Item: 18.4507 | Tusk break: 3  Item: 2  Tuskbreak:Item: 3 | Tusk break: 0.7345151  Item: 0.5896362  Tuskbreak:Item: 0.0003551 *** | X |
| Grasping direction RL: right (1) vs left (0) | Both | Individuals, age class, sex, park | Tusk breaking | Right tusk break: 3  Left tusk break: 1  Both tusks break: 3  No tusk break: 12 | Right tusk break: 9  Left tusk break: 9  Both tusks break: 54  No tusk break: 125 | Tusk break: 1.259 | Tusk break: 3 | Tusk Break: 0.7389 | X |
| Grasping direction LF: lateral (1) vs frontal (0) | Males | Individuals, age class, park | Tusk breaking*Item | Right tusk break: 1  Left tusk break: 1  Both tusks break: 3  No tusk break: 7 | Right tusk break: 10  Left tusk break: 22  Both tusks break: 153  No tusk break: 212 | Tusk break: 4.2006  Item: 1.3252  Tuskbreak:Item: 0.0596 | Tusk break: 3  Item: 2  Tuskbreak:Item: 2 | Tusk break: 0.2406  Item: 0.5155  Tuskbreak:Item: 0.9706 | X |
| Trunk base use: yes (1) vs no (0) | Males | Individuals, age class, park | Tusk breaking | Right tusk break: 3  Left tusk break: 2  Both tusks break: 3  No tusk break: 20 | Right tusk break: 17  Left tusk break: 16  Both tusks break: 109  No tusk break: 259 | Tusk break: 0 | Tusk break: 3 | Tusk break: 1 |  |
| Trunk tip use only: yes (1) vs no (0) | Males | Individuals, age class, park | Tusk breaking*Item | Right tusk break: 1  Left tusk break: 1  Both tusks break: 3  No tusk break: 7 | Right tusk break: 10  Left tusk break: 22  Both tusks break: 153  No tusk break: 217 | Tusk break: 15.0761  Item: 10.6114  Tuskbreak:Item: 4.8963 | Tusk break: 3  Item: 2  Tuskbreak:Item: 2 | Tusk break: 0.001753 **  Item: 0.004963 **  Tuskbreak:Item: 0.086453 | Fig 10 |
| Trunk posture: pinch (1) vs wrap (0) | Both | Individuals, age class, sex, park | Tusk symmetry*Item | Right tusk higher: 1  Left tusk higher: 3  Both symmetric tusks: 17 | Right tusk higher: 7  Left tusk higher: 39  Both symmetric tusks: 326 | Tusk symmetry: 1.0818  Item: 3.0270  Tusksymmetry:Item: 0 | Tusk symmetry: 2  Item: 2  Tusksymmetry:Item: 1 | Tusk symmetry: 0.5822  Item: 0.2201  Tusksymmetry:Item: 0.9981 |  |
| Grasping direction RL: right (1) vs left (0) | Both | Individuals, age class, sex, park | Tusk symmetry*Item | Right tusk higher: 1  Left tusk higher: 3  Both symmetric tusks: 12 | Right tusk higher: 7  Left tusk higher: 16  Both symmetric tusks: 186 | Tusk symmetry: 0.0001  Item: 12.4651  Tusksymmetry:Item: 0 | Tusk symmetry: 2  Item: 2  Tusksymmetry:Item: 1 | Tusk symmetry: 0.999925  Item: 0.001964 **  Tusksymmetry:Item: 0.999756 |  |
| Grasping direction LF: lateral (1) vs frontal (0) | Both | Individuals, age class, sex, park | Tusk symmetry*Item | Right tusk higher: 1  Left tusk higher: 3  Both symmetric tusks: 17 | Right tusk higher: 7  Left tusk higher: 39  Both symmetric tusks: 326 | Tusk symmetry: 2.0654  Item: 0.5461  Tusksymmetry:Item: 0 | Tusk symmetry: 2  Item: 2  Tusksymmetry:Item: 1 | Tusk symmetry: 0.3560  Item: 0.7611  Tusksymmetry:Item: 0.9986 |  |
| Trunk tip use only: yes (1) vs no (0) | Both | Individuals, age class, sex, park | Tusk symmetry | Right tusk higher: 1  Left tusk higher: 3  Both symmetric tusks: 15 | Right tusk higher: 7  Left tusk higher: 38  Both symmetric tusks: 221 | Tusk symmetry: 1.7311 | Tusk symmetry: 2 | Tusk symmetry: 0.4208 |  |
| Trunk posture: pinch (1) vs wrap (0) | Males | Individuals, age class, park | Tusk presence*Item | No tusk: 3  Right tusk: 1  Both tusks: 8 | No tusk: 45  Right tusk: 75  Both tusks: 282 | Tusk presence: 18.9664  Item: 5.2909  Tuskpresence:Item: 0 | Tusk presence: 2  Item: 2  Tuskpresence:Item: | Tusk presence: 7.612e-05 ***  Item: 0.07097  Tuskpresence:Item: | Fig 11 |
| Grasping direction RL: right (1) vs left (0) | Males | Individuals, age class, park | Tusk presence*Item | No tusk: 2  Right tusk: 1  Both tusks: 8 | No tusk: 29  Right tusk: 18  Both tusks: 160 | Tusk presence: 0.4156  Item: 11.6314  Tuskpresence:Item: 0 | Tusk presence: 2  Item: 2  Tuskpresence:Item: | Tusk presence: 0.81236  Item: 0.00298 **  Tuskpresence:Item: |  |
| Grasping direction LF: lateral (1) vs frontal (0) | Males | Individuals, age class, park | Tusk presence*Item | No tusk: 3  Right tusk: 1  Both tusks: 8 | No tusk: 45  Right tusk: 70  Both tusks: 282 | Tusk presence: 3.4943  Item: 0.6492  Tuskpresence:Item: 0 | Tusk presence: 2  Item: 2  Tuskpresence:Item: | Tusk presence: 0.1743  Item: 0.7228  Tuskpresence:Item: |  |
| Trunk base use: yes (1) vs no (0) | Males | Individuals, age class, park | Tusk presence*Item | No tusk: 3  Right tusk: 1  Both tusks: 8 | No tusk: 45  Right tusk: 75  Both tusks: 282 | Tusk presence: 0  Item: 3.8198  Tuskpresence:Item: 0 | Tusk presence: 2  Item: 2  Tuskpresence:Item: | Tusk presence: 1  Item: 0.1481  Tuskpresence:Item: |  |
| Trunk tip use only: yes (1) vs no (0) | Males | Individuals, age class, park | Tusk presence*Item | No tusk: 3  Right tusk: 1  Both tusks: 8 | No tusk: 45  Right tusk: 75  Both tusks: 282 | Tusk presence: 6.7183  Item: 8.0925  Tuskpresence:Item: 0 | Tusk presence: 2  Item: 2  Tuskpresence:Item: | Tusk presence: 0.03476 *  Item: 0.01749 *  Tuskpresence:Item: |  |
| Grasping direction RL: right (1) vs left (0) | Both | Individuals, age class, sex, park | Left tusk size | I: 7  J: 5  K: 2 | I: 56  J: 43  K: 49 | Left tusk size: 1.7804 | Left tusk size: 3 | Left tusk size: 0.6192 |  |
| Grasping direction LF: lateral (1) vs frontal (0) | Both | Individuals, age class, sex, park | Left tusk size | I: 9  J: 8  K: 3 | I: 113  J: 74  K: 83 | Left tusk size: 0.7148 | Left tusk size: 2 | Left tusk size: 0.6995 |  |
| Trunk base use: yes (1) vs no (0) | Males | Individuals, age class, park | Left tusk size*Item | I: 4  J: 2  K: 2 | I: 102  J: 64  K: 116 | Left tusk size: 0  Item: 1.6098  Left tusk size:Item: 0 | Left tusk size: 2  Item: 2  Left tusk size:Item: 3 | Left tusk size: 1  Item: 0.4471  Left tusk size:Item: 1 |  |
| Trunk tip use only: yes (1) vs no (0) | Males | Individuals, age class, park | Left tusk size*Item | I: 4  J: 2  K: 2 | I: 102  J: 64  K: 116 | Left tusk size: 4.3716  Item: 7.7322  Left tusk size:Item: 4.1833 | Left tusk size: 3  Item: 3  Left tusk size:Item: 3 | Left tusk size: 0.22403  Item: 0.05188  Left tusk size:Item: 0.24234 |  |
| Grasping direction LF: lateral (1) vs frontal (0) | Both | Individuals, age class, sex, park | Right tusk size*Item | H: 2  I: 12  J: 7  K: 4 | H: 8  I: 182  J: 119  K: 144 | Right tusk size: 4.2624  Item: 0.7028  Right tusk size:Item: 1.7483 | Right tusk size: 3  Item: 2  Right tusk size:Item: 4 | Right tusk size: 0.2345  Item: 0.7037  Right tusk size:Item: 0.7819 |  |
| Trunk tip use only: yes (1) vs no (0) | Both | Individuals, age class, sex, park | Right tusk size*Item | H: 2  I: 12  J: 7  K: 4 | H: 8  I: 187  J: 119  K: 144 | Right tusk size: 5.8765  Item: 10.4872  Right tusk size:Item: 1.2394 | Right tusk size: 3  Item: 2  Right tusk size:Item: 4 | Right tusk size: 0.117777  Item: 0.005281 **  Right tusk size:Item: 0.871565 |  |
| Trunk posture: pinch (1) vs wrap (0) | Males | Individuals, age class, park | Left tusk opening*Item | Shut tusk: 0  Neutral tusk: 1  Open tusk: 7 | Shut tusk: 0  Neutral tusk: 10  Open tusk: 272 | Left tusk opening: 1.6867  Item: 4.4372  Left tusk opening:Item: 0 | Left tusk opening: 1  Item: 2  Left tusk opening:Item: | Left tusk opening: 0.1940  Item: 0.1088  Left tusk opening:Item: |  |
| Grasping direction RL: right (1) vs left (0) | Males | Individuals, age class, park | Left tusk opening*Item | Shut tusk: 0  Neutral tusk: 1  Open tusk: 7 | Shut tusk: 0  Neutral tusk: 5  Open tusk: 155 | Left tusk opening: 0.6983  Item: 11.9133  Left tusk opening:Item: 0 | Left tusk opening: 1  Item: 2  Left tusk opening:Item: | Left tusk opening: 0.403349  Item: 0.002589 **  Left tusk opening:Item: |  |
| Grasping direction LF: lateral (1) vs frontal (0) | Both | Individuals, age class, sex, park | Left tusk opening*Item | Shut tusk: 3  Neutral tusk: 3  Open tusk: 16 | Shut tusk: 19  Neutral tusk: 60  Open tusk: 297 | Left tusk opening: 8.0315  Item: 0.7878  Left tusk opening:Item: 0 | Left tusk opening: 2  Item: 2  Left tusk opening:Item: | Left tusk opening: 0.01803 *  Item: 0.67443  Left tusk opening:Item: |  |
| Trunk base use: yes (1) vs no (0) | Males | Individuals, age class, park | Left tusk opening*Item | Shut tusk: 0  Neutral tusk: 1  Open tusk: 7 | Shut tusk: 0  Neutral tusk: 10  Open tusk: 272 | Left tusk opening: 3.4809  Item: 6.8857  Left tusk opening:Item: 0 | Left tusk opening: 1  Item: 2  Left tusk opening:Item: | Left tusk opening: 0.06208  Item: 0.03197 *  Left tusk opening:Item: |  |
| Trunk tip use only: yes (1) vs no (0) | Males | Individuals, age class, park | Left tusk opening*Item | Shut tusk: 0  Neutral tusk: 1  Open tusk: 7 | Shut tusk: 0  Neutral tusk: 10  Open tusk: 272 | Left tusk opening: 1.8347  Item: 2.5294  Left tusk opening:Item: 0 | Left tusk opening: 1  Item: 2  Left tusk opening:Item: | Left tusk opening: 0.1756  Item: 0.2823  Left tusk opening:Item: |  |
| Trunk posture: pinch (1) vs wrap (0) | Males | Individuals, age class, park | Right tusk opening*Item | Shut tusk: 0  Neutral tusk: 1  Open tusk: 8 | Shut tusk: 0  Neutral tusk: 10  Open tusk: 347 | Right tusk opening: 1.8347  Item: 2.5294  Right tusk opening:Item: 0 | Right tusk opening: 1  Item: 2  Right tusk opening:Item: | Right tusk opening: 0.1756  Item: 0.2823  Right tusk opening:Item: |  |
| Grasping direction RL: right (1) vs left (0) | Both | Individuals, age class, sex, park | Right tusk opening*Item | Shut tusk: 3  Neutral tusk: 3  Open tusk: 12 | Shut tusk: 10  Neutral tusk: 40  Open tusk: 179 | Right tusk opening: 2.7053  Item: 12.4869  Right tusk opening:Item: 0 | Right tusk opening: 2  Item: 2  Right tusk opening:Item: | Right tusk opening: 0.258558  Item: 0.001943 **  Right tusk opening:Item: |  |
| Grasping direction LF: lateral (1) vs frontal (0) | Both | Individuals, age class, sex, park | Right tusk opening*Item | Shut tusk: 4  Neutral tusk: 4  Open tusk: 15 | Shut tusk: 26  Neutral tusk: 61  Open tusk: 359 | Right tusk opening: 1.8591  Item: 1.3439  Right tusk opening:Item: 0 | Right tusk opening: 2  Item: 2  Right tusk opening:Item: | Right tusk opening: 0.3947  Item: 0.5107  Right tusk opening:Item: |  |
| Trunk base use: yes (1) vs no (0) | Both | Individuals, age class, sex, park | Right tusk opening | Shut tusk: 4  Neutral tusk: 4  Open tusk: 13 | Shut tusk: 26  Neutral tusk: 61  Open tusk: 258 | Right tusk opening: 0 | Right tusk opening: 2 | Right tusk opening: 1 |  |
| Trunk tip use only: yes (1) vs no (0) | Males | Individuals, age class, park | Right tusk opening | Shut tusk: 0  Neutral tusk: 1  Open tusk: 8 | Shut tusk: 0  Neutral tusk: 10  Open tusk: 245 | Right tusk opening: 5.2822 | Right tusk opening: 1 | Right tusk opening: 0.02155 * |  |
